# Molecularly Imprinted Silk Fibroin Nanoparticles

**DOI:** 10.1021/acsami.1c05405

**Published:** 2021-06-30

**Authors:** Alessandra Maria Bossi, Alessio Bucciarelli, Devid Maniglio

**Affiliations:** † Department of Biotechnology, University of Verona, Strada Le Grazie 15, Verona 37134, Italy; ‡ National Council or Research, CNR-Nanotec, Campus Ecotekne - Università del Salento, Via Monteroni, Lecce 73100, Italy; § Department of Industrial Engineering, BIOtech Research Center, University of Trento, Via delle Regole 101, Mattarello, Trento 38123, Italy

**Keywords:** molecularly imprinted polymers, silk fibroin, biomimetics, functional nanoparticles, natural biomaterials

## Abstract

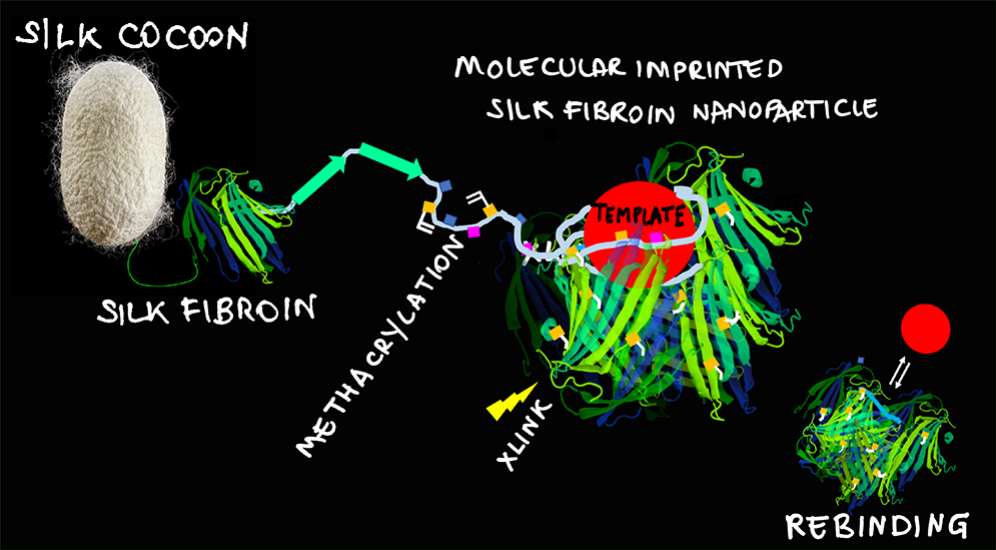

Nanosized biomimetics prepared by the strategy of molecular imprinting, that is, the stamping of recognition sites by means of a template-assisted synthesis, are demonstrating potential as plastic antibodies in medicine, proving effective for cell imaging and targeted therapies. Most molecularly imprinted nanoparticles (MIP-NPs) are currently made of soft matter, such as polyacrylamide and derivatives. Yet, MIP-NPs biocompatibility is crucial for their effective translation into clinical uses. Here, we propose the original idea to synthesize fully biocompatible molecularly imprinted nanoparticles starting from the natural polymer silk fibroin (MIP SF-NPs), which is nontoxic and highly biocompatible. The conditions to produce MIP SF-NPs of different sizes (*d*_mean_ ∼ 50 nm; *d*_mean_ ∼ 100 nm) were set using the response surface method. The stamping of a single, high affinity (*K*_D_ = 57 × 10^–9^ M), and selective recognition site per silk fibroin nanoparticle was demonstrated, together with the confirmation of nontoxicity. Additionally, MIP SF-NPs were used to decorate silk microfibers and silk nanofibers, providing a general means to add entailed biofunctionalities to materials.

## Introduction

The development of functional materials capable of binding target molecules with high affinity and selectivity, *in vitro* and *in vivo*, continues to be of prime interest for medicine, sensing, and bioengineering. In the effort to synthesize receptors mimicking natural ones, the method of molecular imprinting is one of the most conceptually appealing and extensively studied.^1,2^

Molecularly imprinted polymers (MIPs) are prepared by a template-assisted synthesis, which involves the polymerization of functional monomers around a selected molecular template. As a result, cavities with shape and stereochemistry complementary to the template are stamped in the material. At the completion of the polymerization process, the template is removed, leaving the formed cavities free to rebind their target molecule.^3,4^

The high affinity and selectivity for the target molecule are pivotal qualities of a MIP. These are proficiently combined with the physical properties of the constituent polymer (e.g., polymethacrylates and polyacrylamides), which confer to the material qualities such as strain, responsiveness, resistance to solvents, extreme pH, and temperatures, and the possibility of sterilization, processability, and integrability to electronics and devices. Moreover, MIPs are cheap to produce, especially when compared to their biological counterparts.

When synthesized in the form of nanoparticles,^5−7^ MIP-NPs display a close resemblance to antibodies, having a low number of binding sites per NP and a high affinity for the target analyte. Thus, MIP-NPs are ideal for functional interactions with biomolecules, opening applications in molecular diagnosis both at the cell and tissue levels,^8^ for *in vivo* removal of toxins,^5^ for interfering with metastatic proliferation,^9,10^ for protein refolding,^11^ or for acting as targeted prodrugs in contrasting the growth of cancer cells.^12^

Although MIP-NPs stand out because of their potential, their plastic composition might raise concerns, both in medical uses and in terms of environmental safety. Whether biocompatible alternatives to the actual set of materials to be stamped are available is still an open question.

In this respect, Klaus Mosbach,^13^ in a pioneer work demonstrated the modification of the specificity of the active site of an enzyme by its partial unfolding followed by refolding in the presence of a non-natural substrate, setting the principle that a protein can be imprinted. More recently, it was shown how polymers, which are referred to as macromolecular monomers, can be successfully imprinted, including natural materials, such as chitosan and alginates.^14−16^ Frequently, these macromolecular monomers have been chemically modified with reactive double bonds so that the formed MIP can be stabilized by the cross-linking of the polymeric networks, hence improving the physical properties. Indeed, intramolecular cross-linking of polymers can significantly alter the chain arrangement and increase the stiffness of the interior scaffold structure.^1^

The synthesis of MIP-NPs starting from natural polymers could represent the next frontier in nano/materials research, allowing the targeting of additional and specific biological responses, and fully embodying the biomimicry principles. In this framework, a material noteworthy of interest is silk fibroin (SF), a naturally derived polymer, characterized by nontoxicity, biocompatibility, biodegradability, and low thrombogenicity.^17,18^

SF protein from the *Bombyx mori* silkworm has gained considerable attention because it can be processed into a variety of formats, such as films, hydrogels, or foams,^19^ to match different applications, having attractive and tunable mechanical, biological, and optical properties.^20^ Recent reports, in the domain of drug delivery, have also shown the possibility to prepare SF microparticles and even nanoparticles.^21,22^

Here, we report, for the first time, the preparation of imprinted SF nanoparticles (SF-NPs) of controllable nanosizes. The stamping of binding sites in the SF-NPs by molecular imprinting was demonstrated using human serum albumin (HSA)^23^ as a general and widely used model template protein. We studied the affinity, selectivity, and specificity of the formed MIP SF-NPs and confirmed their nontoxicity. At last, in a preliminary experiment, we integrated MIP SF-NPs to silk fibers, which are staked for production of biomedical textiles and for tissue engineering and regenerative medicine (TERM).^24^ MIP SF-NPs decorated raw silk fibers (typical ⌀ ∼ 10–20 μm)^25^ and electrospun silk nanofibers (⌀ ∼ 300 nm)^26^ demonstrated selective binding toward the template, disclosing a possible general method for adding tailored extrafunctions to these biocompatible fibers and opening further the frontiers of nanomaterials for medicine.

## Results and Discussion

SF is a protein well-known for its ability to form entangled fibers by the spontaneous supramolecular assembly of β structures.^27^ From these premises, we defined a strategy to prepare SF biocompatible nanoparticles (SF-NPs) by highly diluting in aqueous solution the SF starting material (to 0.03 or 0.3% w/v) and by allowing the suspensions to stand for 1 h, promoting the formation of separated SF entangled nuclei, each one ideally yielding to a single NP, similar to protocols for the preparation of polymeric NPs.^5,7,11^ With the purpose of forming stable, yet molecularly imprinted, SF-NPs, methacrylated fibroin (SF-MA)^28^ was chosen as the macromolecular monomer.^29^ SF-MA, also known as Sil-MA,^28^ possesses reactive pendant double bonds that permit cross-linking of the formed SF-NPs, hence, their stabilization. The cross-linking of the SF-NPs occurred via UV-induced photo-cross-linking by means of lithium phenyl- 2,4,6-trimethylbenzoyl phosphinate (LAP). Preliminary tests produced positive outcomes (Section 1 of the Supporting Information (SI)).

**Figure 1:**
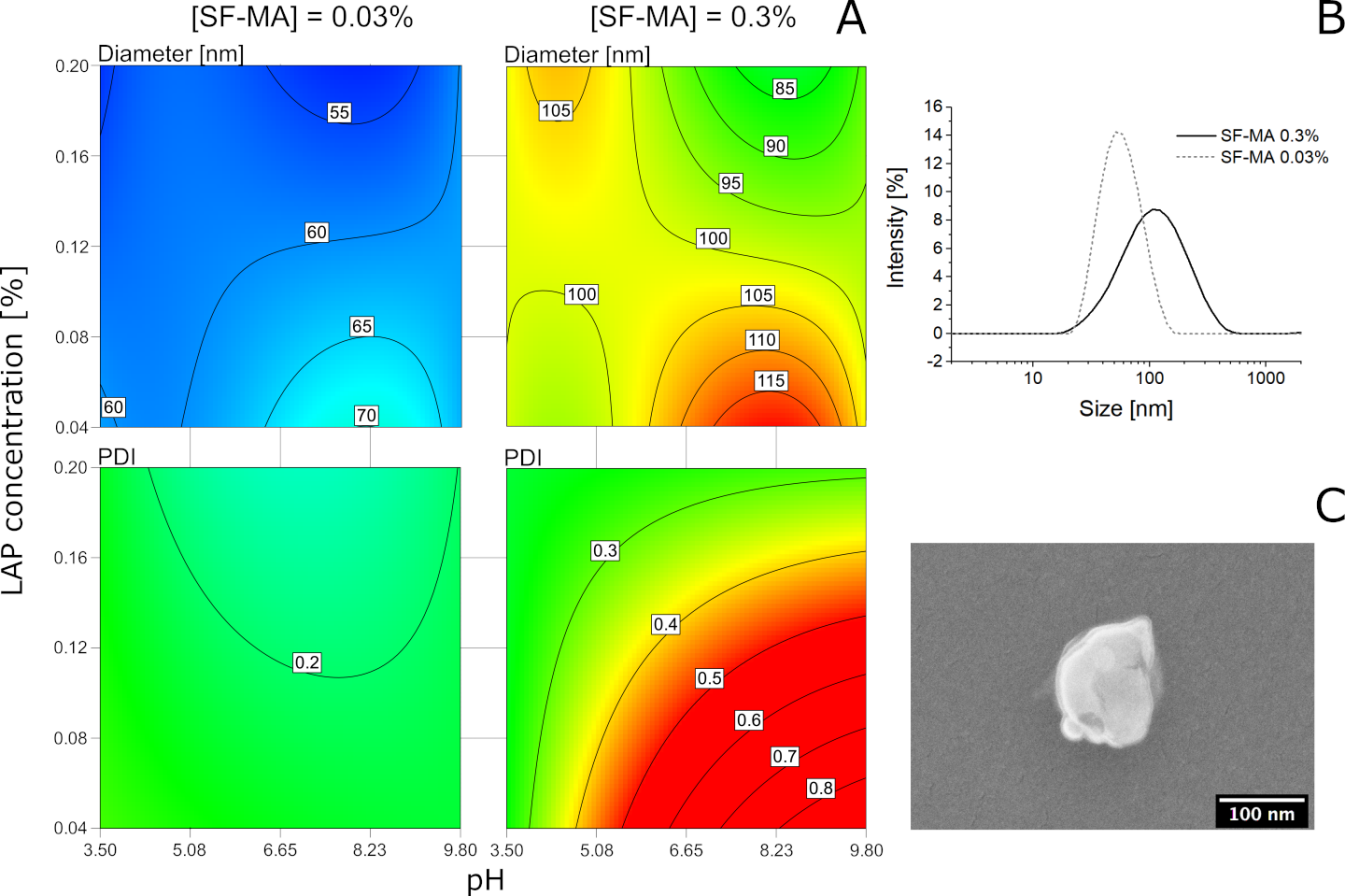
(A) Contour plot of the predicted model of the diameter (top row) and polydispersity index (PDI, bottom row). The model revealed that the SF-MA concentration was a critical parameter for obtaining NPs with a small diameter and a low PDI. (B) Dynamic light scattering (DLS) of albumin-imprinted SF-NPs exhibited an average size of 52.8 ± 0.1 nm (PDI 0.13 ± 0.01) when prepared from 0.03% SF-MA (solid line) and of 94.6 ± 1.3 nm (PDI 0.35 ± 0.01) when prepared from 0.3% SF-MA (dotted line). (C) SEM image of a single SF-NP (0.3% w/v).

Then the response surface method (RSM)^30^ enabled optimization of the SF-NPs synthesis by modeling the nanoparticle’s diameter (*d*) and polydispersity (PDI) on the basis of three process variables: the concentration of SF-MA (factor *A*: 0.03% and 0.3% w/v), the pH of the solution (factor *B*: 3.5, 5.0, 7.4, and 9.8) and the quantity of photoinitiator LAP (factor *C*: 0.04% and 0.2% w/v) (Figure 1; details in Section 2, SI). The RSM allowed exploration of the correlation among the three chosen variables, finding the optimal combinations to minimize the particle’s size dispersion, evaluated through the PDI values. The ANOVA tables (Section 2, SI) revealed that the considered variables significantly affected (with *p* < 0.0001) both the mean particle diameter and the PDI. Interestingly, the significance of several second-order terms and of one third-order term, not detectable by a one-factor-at-a-time method, could be distinguished. In particular, for both *d* and PDI, all the second-order terms (*A*·*B*, *A*·*C*, *B*·*C*, *A*^2^·*B*) and the third-order term (*A*·*B*·*C*) were significant, indicating that all the considered factors are interacting with each other. In addition, in the case of *d* the term *A*^3^*B* and in the case of PDI the term *A*^2^·*C* were significant. The diameter data were well fit by a cubic model, whereas a quadratic model was sufficient to fit the PDI data. As can be clearly seen in the contour plots of Figure 1, these high-order terms resulted in a complex trend for the diameter, with several local minima and maxima. However, the concentration had the greater impact on *d*: the lower SF-MA concentration yielded to smaller nanoparticles. The PDI was influenced too by the concentration, as the low SF-MA concentration (0.03%) ensured a low PDI regardless of the pH and LAP concentration. Yet, at a higher SF-MA concentration (0.3%), the PDI proved to be dependent also on other factors, as an LAP lower than 0.2% and pH higher than 4.7 did not allow a sufficiently narrow distribution (PDI < 0.4) to be obtained.

As a next step, we attempted the imprinting of the SF-NPs. The protein human serum albumin (HSA) was chosen as a template.^23^ HSA (3 nmol) was added to the SF-MA synthetic mixtures (0.03% and 0.3% w/v; *V* = 4 mL), in the presence of LAP 0.2% w/v. After photo-cross-linking, albumin imprinted SF-NPs (MIP-alb SF-NPs) with a *Z*_average_ of respectively 52.8 ± 0.1 (PDI 0.13 ± 0.01) and 94.6 ± 1.3 nm (PDI 0.36 ± 0.02) were obtained (Figure 1B). The sizes of the imprinted SF-NPs were coherent with the synthetic process in the absence of a template, confirming that the template did not perturb the system. The estimated mean molecular masses of the SF-NPs were 7 and 21 MDa, respectively, for SF-MA 0.03% and 0.3% w/v (static light scattering, Section 4, SI). The yield of the synthetic reaction, calculated from the dry weight of the SF-MA starting material compared to the dry weight of the formed SF-NPs, was >90%, accounting for the efficiency of the proposed synthetic method. The zeta potential of the SF-NPs over pH was also studied (Figure S5.1). At last, regenerated silk fibroin suspensions tend to evolve over time. In our case, SF-NPs in buffer were stable for 72 h.

**Figure 2:**
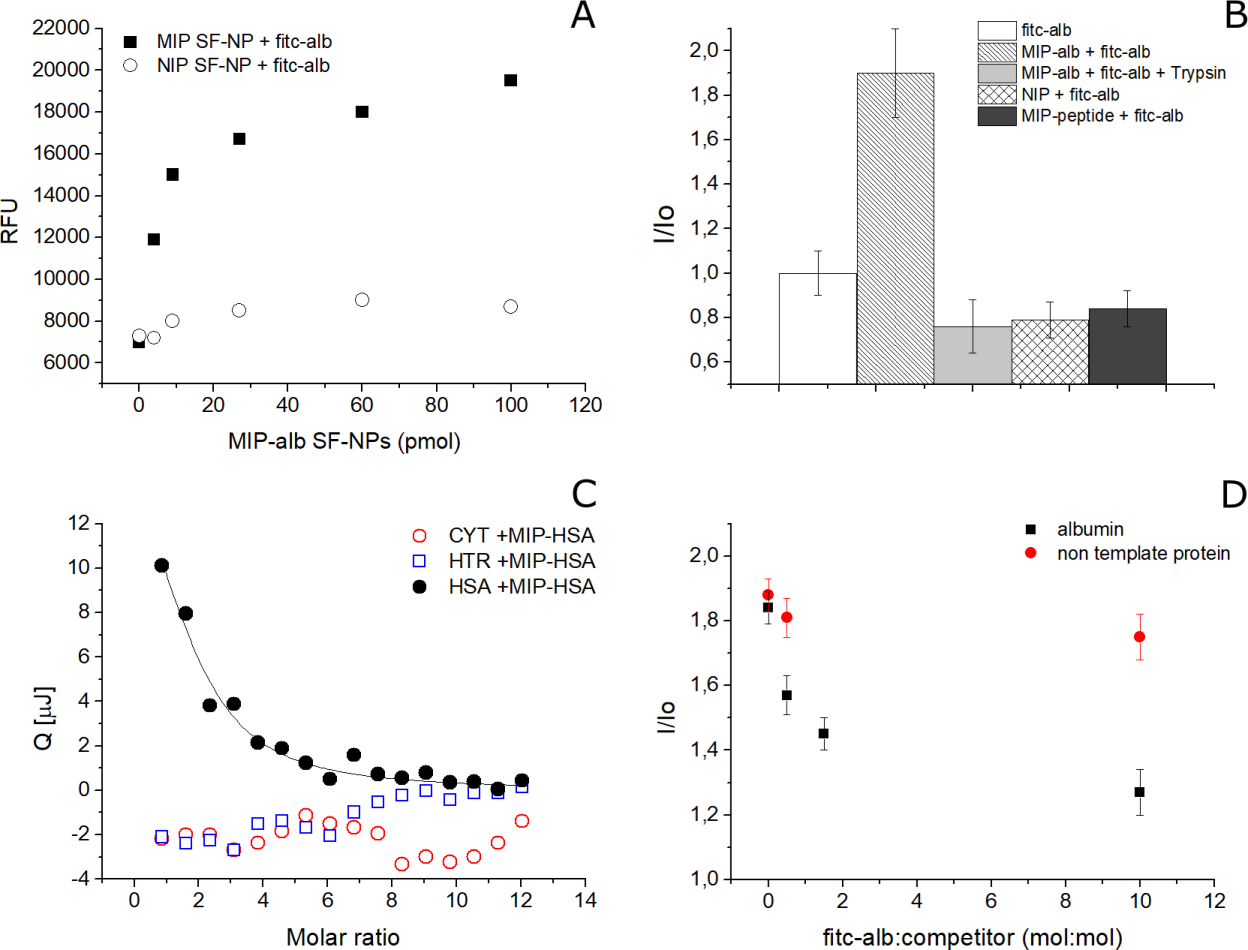
(A) MIP-alb SF-NPs (solid squares, *n* = 3, Stvd 10%) showed to rebinding of the template HSA with a saturation course; in contrast, control SF-NPs (NIP; open circles, *n* = 3, Stdv 7%) showed nonspecific interaction. (B) Binding assay in fluorescence expressed as *I*/*I*_o_. (C) Nanocalorimetric measurements showed MIP-alb SF-NPs were selective for the template (HSA, solid circles), whereas nontemplate proteins, such as cytochrome c (red open circles) and human transferrin (blue open squares), did not show interaction (*n* = 3, Stdv 5%). (D) Competitive assay in fluorescence expressed as *I*/*I*_o_: MIP-alb SF-NPs bound to fitc-alb amd challenged with increasing quantities of nonfluorescent albumin (solid squares) showed progressive displacement of the binding, whereas when there was a challenge with a nontemplate protein (transferrin; red circles), no competition was observed.

Prior to investigation of the binding abilities of the MIP-alb SF-NPs, the template was removed from the binding cavities (Section 6, SI). Next, the MIP-alb SF-NPs were tested for their affinity, selectivity, and specificity. The specificity of the binding of MIP-alb SF-NPs for the template was investigated using a fluorescein-labeled HSA (fitc-alb).^31^ fitc-alb (30 pmol) was incubated in the presence of increasing concentrations of MIP-alb SF-NPs or of nonimprinted SF-NPs (Figure 2A; details in Section 7, SI). A saturation profile was observed for the binding of fitc-alb to MIP-alb, whereas control SF-NPs showed a nonspecific course, supporting the success of the imprinting process. As a control, the endogenous fluorescence of MIP-alb SF-NPs was far lower than the signal emitted by fitc-alb (30 pmol) (Figure S7.1). The binding of fitc-alb to the MIP-alb SF-NPs resulted in a fluorescence enhancement, *I*/*I*_o_ (Figure 2B, Figure S7.2). When the binding conditions for fitc-alb to MIP-alb turned unfavorable and promoted the disruption of the complexes (addition of Trizma base to the incubation solution, yielding to an extreme pH of 10), we observed a drop in the emitted fluorescence (Figure S7.1). Equally low was the fluorescence of fitc-alb (30 pmol) both in the presence of nonimprinted NIP SF-NPs and in the presence of MIP SF-NP prepared by using a peptide template nonrelated to albumin, reinforcing the hypothesis that specific albumin imprinted binding sites were stamped through the SF imprinting process (Figure 2B, Figure S7.2). At last, the addition of trypsin to MIP-alb/fitc-alb complexes, supposedly digesting fitc-alb, resulted once more in a drop in the emitted fluorescent signal (Figure 2B, Figure S7.2).

Then the affinity and selectivity of the MIP-alb SF-NPs were investigated by means of isothermal titration calorimetry.^32,33^ MIP-alb SF-NPs (330 nM) were suspended in PB buffer and titrated with increasing quantities of the template protein (HSA, 3 μM; titrations of 3 μL, *V* = 50 μL) or of nontemplate proteins (human transferrin, 3 μM; cytochrome c, 3 μM; titrations of 3 μL, *V* = 50 μL). The heats associated with the protein-to-nanoparticle interactions were recorded over time, integrated and plotted as a function of the molar ratio MIP-alb SF-NP/protein (Figure 2C; details in Section 8, SI). On the basis of the distribution of the integrated heats over the molar ratio between titrant and titrand, the fitting of the experimental data was performed by means of an independent single-binding site model, which is a widely accepted model to estimate an averaged dissociation constant and averaged number of binding sites.^34^ As the synthesis of the MIP SF-NPs was not followed by a downstream affinity selection process,^35^ to pick just the nanoparticles bearing full imprints, a statistical distribution of binding sites was expected; MIP SF-NPs having high affinity mingled with particles with scarce or no binding site. Fitting the HSA to MIP-alb SF-NP calorimetric interaction data with the independent site model resulted in a dissociation constant *K*_D_ = 57.0 ± 2.7 × 10^–9^ M. The stoichiometry of the interaction was 1.25 ± 0.54 mol of HSA/mol of NPs, indicating the proposed synthetic process permits stamping MIP SF-NPs with a quasi-single high-affinity binding site per particle, on average. Yet, confirmation over the formed number of printed binding sites per nanoparticle, for example, by NMR,^36^ and more generally over the formation of the SF-NP around the template would be desirable to further master the tailoring of recognition in Sil-MA NPs.

The MIP-alb SF-NPs proved selective for their template. Indeed, no binding was observed when MIP-alb SF-NPs were titrated with nontemplate proteins (Figure 2C). The selectivity of MIP-alb SF-NPs was also confirmed in a competitive assay (Figure 2D). We monitored the fluorescence of MIP-alb SF-NPs bound to fitc-alb (30 pmol) when challenged with increasing quantities of nonfluorescent HSA (30–300 pmol). The decrement in emitted fluorescence appeared to correlate to the quantity of added HSA, suggesting a competitive displacement of the fitc-alb by its nonfluorescent analogousness (Figure S7.3). A dissimilar displacement was observed when the competitor was the non-template-related protein transferrin (Figure 2D).

Then MIP-alb SF-NPs suspensions were tested for toxicity to assess their cytocompatibility. Almost confluent mouse embryonic fibroblasts (NIH 3T3) were exposed to a highly concentrated SF-NP-containing medium (0.25 and 1.5 mg/mL) for 24 h and showed a release of lactate dehydrogenase (LDH) levels compatible with the negative controls (not exposed to treatment) (Figure S9.1). This result fully confirmed the noncytotoxicity of the SF-NPs, even at elevated concentrations. So far, the results demonstrated the success in synthesizing highly homogeneous, high affinity, selective, and cytocompatible MIP-SF nanoparticles.

The MIP SF-NPs, being nontoxic single binding site recognition units, display ideal characteristics to build more complex macromolecular architectures, with a foreseen impact on the landscape of next-generation smart wearables and *in vivo* implantable medical devices, like patches for wound healing or scaffolds for TERM applications.^37^ Indeed, the combination of molecular imprinting with fibroin bioactivity and degradability would open a new spectrum of uses, enforcing possibilities in wound healing patches and functional scaffolds.^38^

**Figure 3:**
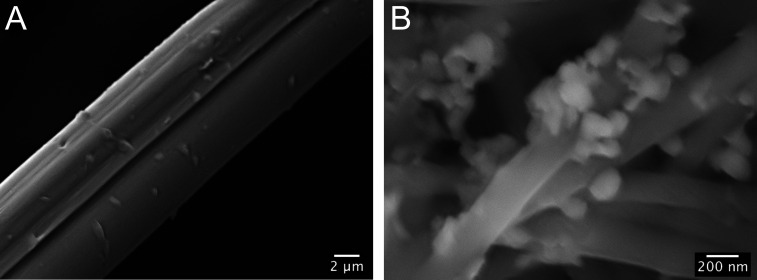
SEM images of MIP SF-NPs decorated: (A) silk microfibers and (B) electrospun silk nanofibers.

As a preliminary exploration, we verified the possibility of preparing functional fabrics by integrating the MIP SF-NPs to silk fibers. We selected fibers of different dimensions: raw silk fibers, with typical micrometric diameters and electrospun nanofibers. The silk microfibers were recovered from raw cocoons and treated in washing baths to remove sericin coating, whereas the nanofibers were fabricated starting from a SF solution on formic acid and electrospun to obtain a nonwoven tissue with a fiber dimension of 300 ± 75 nm. MIP-alb SF-NPs were coupled to both the micro- and nanofibers by means of the carbodiimde/succinimide chemistry.^39^ The decoration of the fibers with SF-NPs was confirmed by scanning electron microscopy (SEM; Figure 3).

**Figure 4:**
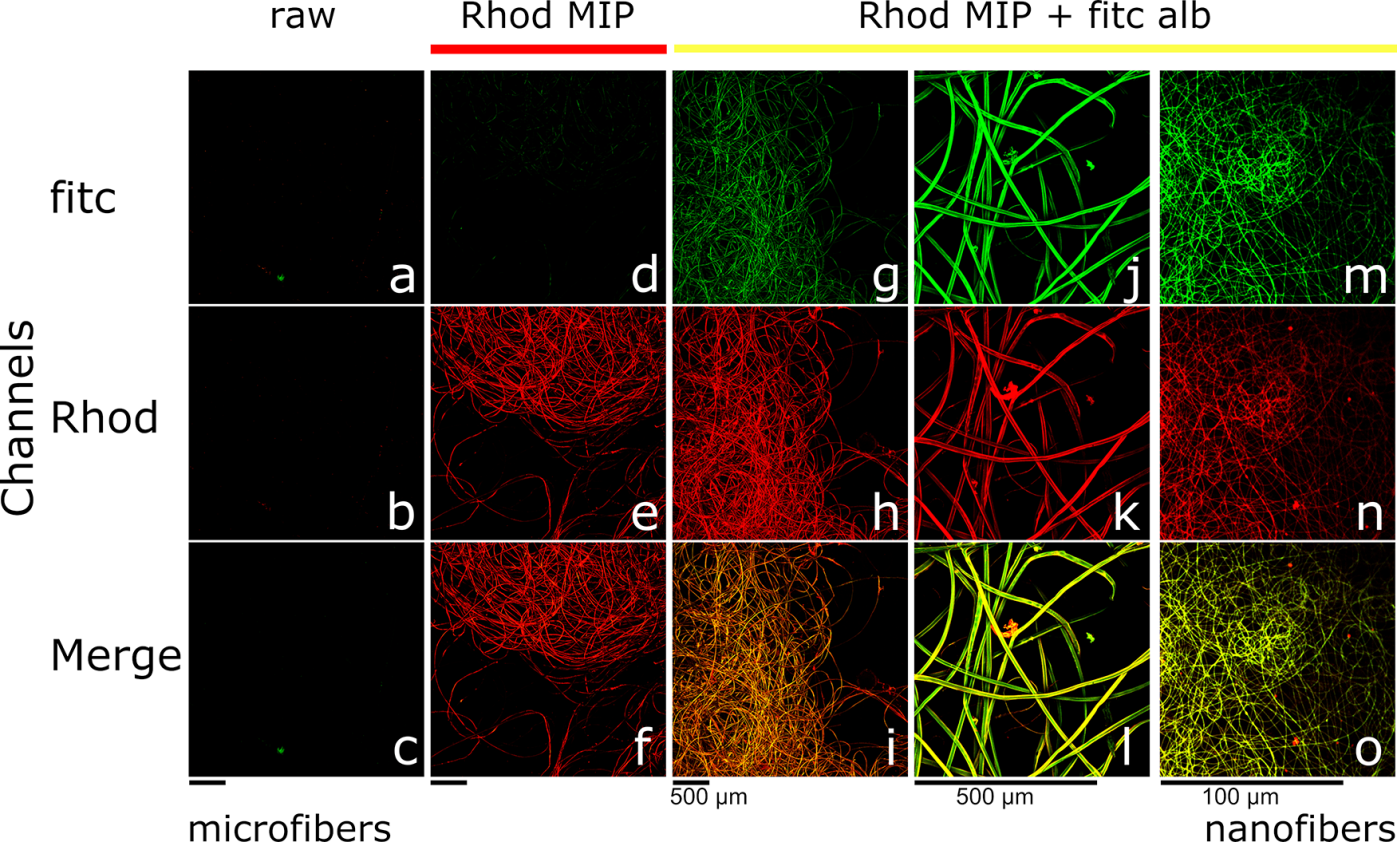
Upper panels (FITC) report the emissions at λ_em_ = 595/50 nm; the middle panels (Rhod) report the emissions at λ_em_ = 525/50 nm; lower panels (Merge) report the combined emissions of fluorescein and rhodamine. Controls, raw silk fibers, showed no emissions, accounting for no endogenous fluorescences (a–c). Silk fibers coupled to rhodamine-MIP SF-NPs showed red emission, confirming a uniform decoration (d–f). Silk fibers decorated with rhodamine-MIP SF-NPs (magnification 4×) and challenged with fitc-alb showed rebinding of fitc-alb (g–i) and at increased magnification (20×) showed the colocalization of the green and red fluorescent emissions (j–l). Electrospun silk nanofibers decorated with rhodamine-MIP SF-NPs and challenged with fitc-alb demonstrated binding of fitc-alb (m–o) (magnification 100×).

To test the functional abilities of MIP SF-NPs fabrics, we prepared rhodamine-MIP-alb SF-NPs and rhodamine-SF-NPs, as a control, that were next coupled to both micro- and nanofibers. Results, imaged by confocal microscopy, are shown in Figure 4. Controls, raw silk microfibers, did not exhibit endogenous fluorescence (Figure 4a–c). Upon decoration, rhodamine MIP-alb SF-NPs (λ_exc_ = 561.6 nm) silk microfibers showed a characteristic and homogeneous fluorescent signal (λ_em_ = 595/50 nm), suggesting a uniform functionalization (Figure 4e,f).

To test the functional response of MIP NP-decorated microfibers, we incubated (2 h) fibers with fluorescein-labeled HSA (fitc-alb; λ_exc_ = 488 nm, λ_em_ = 525/50 nm), followed by washings to remove nonspecific binding. When the fibers were imaged by confocal microscopy, no endogenous fluorescence was emitted by rhodamine MIP-fibers excited at λ_exc_ = 488 nm in the absence of fitc-alb (Figure 4d), whereas fitc-alb incubated fibers (Figure 4g,j) demonstrated emission at λ_em_ = 525/50 nm, confirming the rebinding of the target protein. The considerable overlap of the rhodamine-MIP SF-NP and the fitc-alb signals let us hypothesize that the binding occurred specifically on the MIPs (Figure 4i,l). This was confirmed by the absence of fitc-fluorescence emissions in the case of control nonimprinted SF-NP fibers incubated with fitc-alb (Figure S10.1). At last, we also present the decoration of silk nanofibers with MIP SF-NPs (Figure 4n); the colocalization of the fitc-alb (Figure 4m) and of the rhodamine-MIP NPs (Figure 4o) signals proved the decorated nanofibers gained tailored functionality.

## Conclusions

In summary, we developed an original protocol using methacrylated silk fibroin as a starting material to prepare biocompatible fully protein molecularly imprinted nanoparticles (MIP SF-NPs). MIP SF-NPs demonstrated selective and specific binding for their target analyte together with nontoxic, thus embodying the concept of tailor-made functional biomimetics. As a notable advantage, imprinting silk fibroin involves the whole range of amino acid side chains present on the fibroin backbone to potentially interact with the template. Relying on such a pool of different chemical functionalities is expected to maximize the template/monomer molecular pairing during the imprinting process, ultimately stamping high-affinity binding sites, in close similarity to Nature. The nanomolar affinity observed in our case fully supports the hypothesis. In contrast, the use of other natural polymers, such as polysaccharides (chitosan, alginate, etc.), whose structures are composed of repetitions of the same monomer unit, strongly restrict the portfolio of functional groups available for the imprinting. Moreover, MIP SF-NPs were demonstrated to easily and successfully couple to silk microfibers and to silk electrospun nanofibers. The example provided by these all-silk MIP-NPs fiber superstructures holds the potential to open a general path to add specific extrafunctionalities to biomaterials, with a foreseen impact on biopolymer manufacturing and TERM. As a final consideration, MIP SF-NPs are greener than most of the polymeric materials generally employed for imprinting, thus offering complementary advantages to the current set of nanomaterials.

## Experimental Section

### Silk Fibroin Preparation

Silk cocoons were imported from Thailand (Chul Thai Silk Co., Phetchabun, Thailand). Extraction and purification of silk fibroin was conducted using an adapted version of a well-known protocol.^40^ Briefly, for separation of silk fibroin from silk sericin, *Bombyx mori* silk cocoons were cut into small pieces and placed in a 0.01 M hot bath of sodium carbonate (Na_2_CO_3_, Sigma-Aldrich) for 1 h, followed by a second bath of sodium carbonate with a concentration of 0.003 M for 1 h. The resultant silk fibroin, progressively taken at room temperature, was carefully rinsed three times using ultrapure water and then dried for 2 days. The degummed dry fibroin was later dissolved in a water solution of lithium bromide (9.3 M) at 20% (w/v) concentration for 4 h at 65 °C (step 1). The solution was dialyzed in Slide-A-Lyzer Dialysis Cassettes with 3.5 kDa MWCO (Thermo Fisher Scientific, Waltham, MA, USA) in distilled water for 4 days to remove LiBr residues with regular water changes. After dialysis, the solution was filtered to remove silk fibroin solid agglomerates and freeze-dried (step 2).

### Methacrylation Procedure

Fibroin-MA was prepared following a protocol described elsewhere.^41^ Briefly, glycidyl methacrylate (GMA, Sigma-Aldrich) was added to the fibroin/LiBr/water solution (paragraph 1, step 1), 1 mL/4 g of fibroin. The solution was then stirred at 65 °C for 4 h to allow the conjunction reaction. For removal of the salt and the unreacted GMA, the resulting Sil-MA solution was dialyzed for 4 days against water using a 3.5 kDa dialysis tube. The solution concentration in mg/mL was checked using a spectrophotometer (BioSpectrometer basic, Eppendorf), evaluating the intensity of the A_280_ protein peak (λ = 280 nm).

### Response Surface Method

The entire statistical analysis was performed with the use of the programming language R^42^ following the statistical strategy described in previous works.^28,43,44^ An initial comparison by verifying the presence of a significant difference among the different groups has been done by using the analysis of variance (ANOVA) followed by a Tukey multicomparison test. The model goodness-of-fit was evaluated by the coefficient of determination (*r*^2^) and plotted as a matrix. The level of significance was assigned as follows: *p* ≤ 0.1 (•), *p* ≤ 0.05 (*), *p* ≤ 0.01 (**), *p* ≤ 0.001 (***). A response surface methodology (RMS) was adopted to model the empirical equations to correlate the considered factors to the yields. In this case, we considered three continuous factors: the pH of the solution (four levels, factor *A*), the concentration of the LAP photoinitiator (two levels, factor *B*), and the concentration of the methacrylated fibroin (two levels, factor *C*). As yields, we considered the mean dimension of the particles and the index of polydispersity. Trials are listed in Table S2.1. Equation 1 represents the complete model. An ANOVA test followed by a Tukey multicomparison was conducted to verify the significance of each term of the reported equation. Only the terms with a significant effect (*p* ≤ 0.05) were included in the model. The *F* function was chosen to normalize the model residues and to make them patternless. The whole model was considered significant with *p* ≤ 0.05.
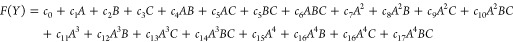
1

### Synthesis of SF-NPs

The SF-MA concentration was adjusted to 0.3% or to 0.03% w/v in Milli-Q water, or in the following buffers: 10 mM acetate Tris, pH 3.5; 10 mM MES, pH 5.0; 10 mM Tris HCl, pH 7.0; 10 mM CAPSO, pH 9.8. The photoinitiator lithium phenyl-2,4,6-trimethylbenzoylphosphinate (LAP, Sigma-Aldrich) was added at the final concentration of 0.2% or 0.04% v/w and photopolymerized for 10 min under UV light, λ = 365 nm (UView Mini Transilluminator, BioRad, Hercules, US). Samples were prepared at least in triplicate.

### Synthesis of MIP SF-NPs

The SF concentration was adjusted to 0.3% or to 0.03% w/v in 10 mM acetate Tris, pH 3.5, or in 5 mM PB, pH 7.0, buffer, in the presence of the print molecule (3 nmol of human serum albumin, Sigma-Aldrich). The final volume was 4 mL. LAP was added at the final concentration of 0.2% or 0.04% v/w and polymerized as reported above. Rhodamine-MIP-alb SF-NPs were prepared in the same manner, but with the addition of 40 μL of acryloxyethyl thiocarbamoyl rhodamine B (Sigma-Aldrich) dissolved at 0.02% w/v in DMSO. At the end of the cross-linking process, the print molecule was removed by the addition of Trizma free base to the NPs suspension to reach a pH of 9.7 for 1 h, and then the MIP SF-NPs were dialyzed with Milli-Q water 4 × 3 L under stirring. Alternatively, for removal of the protein template, the enzyme trypsin (80 μg) was added to the NPs for 1 h at room temperature and at the pH of 8.0, followed by acidification of the solution and dialysis. Protein removal was controlled by SDS-PAGE electrophoresis.

### Scanning Electron Microscopy (SEM)

Images were obtained using a Supra 40 (Zeiss, Germany) field-emission scanning electron microscope. Images were acquired in secondary electron mode at 5 kV. For the SEM analyses, SF-NPs were suspended in a water–ethanol solution at 1 mg/mL; the dispersion was further diluted 10 and 100 times in deionized Milli-Q water. The dispersion was deposited onto a monocrystalline gold-coated silicon chip (120 nm thickness) and freeze-dried to remove water, preserving a tridimensional structure.

### Dynamic Light Scattering

The size distribution and polydispersity index (PDI) were determined by dynamic light scattering (DLS) using a Zetasizer Nano ZEN3600 (Malvern Instruments Ltd., Worcestershire, UK) equipped with a 633 nm He–Ne laser. SF-NPs were dispersed in filtered deionized water at 1 mg/mL and filtered 0.22 μm prior to measure. The material refractive index (RI) was 1.490 and the absorption value was 0.01; the dispersant RI was 1.332, for the viscosity was 0.89 cP as reported by the Zetasizer v.6.32 software (Malvern Instruments Ltd., Worcestershire, UK). The temperature was set at 298 K, and a detection angle of 173° was used. Measurements were collected in triplicate.

### Static Light Scattering

A Zetasizer Nano ZEN3600 (Malvern Instruments Ltd., Worcestershire, UK) equipped with a 633 nm He–Ne laser was used to measure the number-average molar mass (*M*_n_) of the SF-NPs. Latex monosize standards 15–153 nm (Idc Spheres Portland, UK) were used to calibrate the system. SF-NPs were diluted to five concentrations in the range 5–0.14 mg/mL and measured. Raw data were analyzed by the Debye plot, *KC*/*R*θ versus particle concentration, where *K* is the optical constant, *C* is the particle concentration, and *R*θ is the sample Rayleigh ratio; the linear fit intercept corresponds to 1/*M*_n_. A particle refractive index increment (d*n*/d*C*) of 0.17 mL/g and a spherical particle shape (*R*_g_ = 0.740 Rh) were considered. The RI, viscosity, absorption values, and the Rayleigh ratio were provided by the Zetasizer v.6.32 software (Malvern Instruments Ltd., Worcestershire, UK); the refractive index increment (d*n*/d*C*) was found in the American Polymer Standards Corporation.

### Yield of the Synthesis

The synthetic yield was calculated knowing the initial quantity of SF-MA in each synthetic batch (in triplicate). Each polymerized batch of SF-NPs was dialyzed in water, freeze-dried, and weighed. The results indicated the yield was >90%.

### Zeta Potential

The zeta potential was measured on a Zetasizer Nano ZS instrument (Malvern Instruments, Worcestershire, UK) equipped with a 633 nm He–Ne and a universal dip cell (ZEN1002). SF-NPs (from 0.3% w/v SF-MA) were suspended at 1 mg/mL in 10 mM PB, pH 7.0. The zeta potential was estimated by applying the Smoluchowski model and by using the buffer viscosity and RI values (RI = 1.330 and viscosity = 0.8872 cP). Measurements were performed in triplicate.

### Isothermal Titration Nanocalorimetry

A Nano ITC Standard Volume (TA Instruments, Newcastle, US) with a low volume fixed gold cell (190 μL) was used. MIP SF-NPs were suspended in 50 mM PB, pH 7.4, to a final concentration of 300 nM. All the tested proteins, the template albumin and the nontemplates human transferrin and cytochrome c, were solvated in PB to the final concentration of 3 μM. All samples were degassed under vacuum for 5 min. The reference cell was filled with 200 μL of degassed PB and the sample cell was filled with an equal volume of MIP SF-NPs, whereas 50 μL of protein was loaded in the syringe. Each ITC experiment consisted of 16 injections of 3 μL at an interval of 300 s from each other. Experiments, performed in triplicate, were conducted at 25 °C. Data were fitted with the independent site model using the Nano Analyze Software v. 3.4.0 (TA Instruments, New Castle, DE), according to the manufacturer: Bound = (−*b* – Sqrt (*b* * *b* – 4 * *a* * *c*))/(2 * *a*); Ka_value = 1/*K*_d_; the quadratic constants *a*, *b*, and *c* were defined as follows: *a* = Ka_value; *b* = −Ka_value * (MolesSyringe (iteration) + MolesCell (iteration) * *n*) – CellVolume (iteration/1e6); *c* = Ka_value * MolesSyringe (iteration) * MolesCell (iteration) * *n*. The enthalpy (Δ*H*°) was calculated from Heat = 1e9 * (Bound – Old bound) * *dh*; and free energy variations (Δ*G*°) were calculated from *K*_d_ and Δ*H*°.

### Binding to MIP or to NIP SF-NPs

fitc-alb was used to test the binding of MIP-alb SF-NPs. Measurements were performed in triplicate on 96 Flat Bottom Black Polystyrene microtiter plates (ThermoScientific, Germany). Wells were loaded with increasing quantities (0.5–100 pmol) of MIP-alb SF-NPs, or with control nonimprinted SF-NPs (NIP), or with MIP SF-NPs prepared using a peptide nonrelated to albumin as a template and incubated with fitc-alb (30 pmol) in PB 50 mM, pH 7.4, supplemented with 0.05% Tween-20 (*V*_tot_ = 1 mL). As a further control, MIP-alb SF-NPs bound to fitc-alb were then treated with Trypsin (80 μg) to digest fitc-alb. The excitation was at λ_exc_ = 488 nm and emission was recorded in the range 514–540 nm. Maximum λ_em_ was at 524 nm.

### Competitive Binding

MIP SF-NPs (27 pmol) were incubated in triplicate for 30 min in 50 mM PB, pH 7.4, supplemented with 0.05% Tween 20 (*V*_tot_ = 250 μL) in the presence of the sole fitc-alb (30 pmol) and then challenged with increasing quantities of nonfluorescent albumin (30–300 pmol). As an alternative, the nonrelated protein human serum transferrin was used as a competitor. Spectra were recorded as detailed above.

### Cell Toxicity

The potential cytotoxicity of SF-NPs was evaluated using lactate dehydrogenase (LDH) assay (TOX7 In Vitro Toxicology Assay Kit, Sigma-Aldrich) following ISO 10993 standard. Mouse embryonic fibroblasts (NIH 3T3) were seeded in 96-well TCP and cultured in a standard medium until about 70% confluence. The nanoparticles were then suspended into the medium with different concentrations (1.5 and 0.25 mg/mL).

The cytotoxic effect was measured on the basis of the amount of LDH released by cells after 24 h of exposure to the surface-contacting medium. The positive control for cytotoxicity was constituted by fully lysate cells after exposure to 0.5% Triton X. Negative control was obtained from cells in a reduced medium without surfactant. The LDH level was evaluated by light absorbance at 490 nm (Tecan Spark 10 M). Assays were carried out in quintuplicate per each test condition. The noncytotoxicity of the prepared biocompatible selective nanomaterial was tested following ISO 10993 standard, using NIH 3T3 cell line expanded with the respective standard medium and evaluated at about 70% confluence. Cell death percentage was evaluated measuring lactate dehydrogenase released in the medium by cells fed with medium containing 0.25 and 1.5 mg/mL concentration nanoparticles suspension and then compared with negative control (nontreated cells, as reference for noncytotoxic material) and with positive control (all cells dead, as reference for totally cytotoxic material). One-way ANOVA followed by Tukey post hoc test with significance threshold set to 0.05 revealed no significant difference among samples exposed to nanoparticles and negative control.

### Functional Fabrics

MIP SF-NPs were used to decorate different silk fibroin fibers: raw microfibers and nonwoven electrospun fabrics. Raw microfibers were obtained after a degumming procedure (see “Silk Fibroin Preparation”). Nonwoven fabrics were obtained starting from solid fibroin obtained after freeze-drying of fibroin water solution after dialysis (paragraph 1, step 2), with it being dissolved in formic acid (10% w/v) and electrospun onto an aluminum foil placed at a 15 cm distance under 15000 kV potential. The nonwoven fabric was then dipped into methanol to induce β-sheet transition and then rinsed several times with water. The obtained electrospun fibers were analyzed by SEM to evaluate the uniformity of the fibers morphology. The average fiber diameter was estimated with Fiji software^45^ using a ridge detection plugin.

### Decoration of Silk Fibers with MIP SF-NPs

Decoration with rhodamine-MIP SF-NPs and NIPs (200 μL) was performed separately on fibroin microfibers and on electrospun nanofibers by the addition of 100 μL of 1-ethyl-3-(3-(dimethylamino)propyl) carbodiimide (200 mM) and 100 μL of *N*-hydroxysuccinimide (50 mM) and 100 μL of MES buffer (50 mM at pH 5.0). The coupling reaction was let to proceed for 2 h, followed by washings in 20 mM PB, pH 7.4.

### Binding of alb-fitc on Decorated Silk Fibers

Rhodamine-MIP and NIP decorated silk fibers were incubated with 1.2 nmol of alb-fitc in 200 μL of 50 mM PB, pH 7.4, for 1 h. Then micro- and nanofibers were washed three times with the same PB buffer supplemented with 0.05% Tween 20. The fibers were imaged by confocal microscopy (Nikon A1), scanning with a 488 and 561.6 nm laser and collecting fluorescence emission through 525/50 (rhodamine) and 595/50 nm (fitc) band-pass filters, at 4×, 20×, and 100× magnifications.

## Supporting Information Available

Dynamic light scattering of nonmethacrylated silk fibroin nanoparticles; preliminary experimental evidence over the formation of SF-NPs; surface response method; scanning electron microscopy images; static light scattering for the estimation of molecular weight; zeta potential; template removal; binding of template to MIP SF-NPs; isothermal titration nanocalorimetry; cell toxicity; functional silk fibers decorated with MIP SF-NPs (PDF)

## References

[ref1] Chen J.; Garcia E. S.; Zimmerman S. C. Intramolecularly Cross-Linked Polymers: From Structure to Function with Applications as Artificial Antibodies and Artificial Enzymes. Acc. Chem. Res. 2020, 53 (6), 1244–1256. 10.1021/acs.accounts.0c00178. 32441091

[ref2] BelBruno J. J. Molecularly Imprinted Polymers. Chem. Rev. 2019, 119 (1), 94–119. 10.1021/acs.chemrev.8b00171. 30246529

[ref3] Wulff G.; Sarhan A. Über Die Anwendung von Enzymanalog Gebauten Polymeren Zur Racemattrennung. Angew. Chem. 1972, 84 (8), 364–364. 10.1002/ange.19720840838.

[ref4] Andersson L.; Sellergren B.; Mosbach K. Imprinting of Amino Acid Derivatives in Macroporous Polymers. Tetrahedron Lett. 1984, 25 (45), 5211–5214. 10.1016/S0040-4039(01)81566-5.

[ref5] Hoshino Y.; Kodama T.; Okahata Y.; Shea K. J. Peptide Imprinted Polymer Nanoparticles: A Plastic Antibody. J. Am. Chem. Soc. 2008, 130 (46), 15242–15243. 10.1021/ja8062875. 18942788

[ref6] Canfarotta F.; Poma A.; Guerreiro A.; Piletsky S. Solid-Phase Synthesis of Molecularly Imprinted Nanoparticles. Nat. Protoc. 2016, 11 (3), 443–455. 10.1038/nprot.2016.030. 26866789

[ref7] Ambrosini S.; Beyazit S.; Haupt K.; Tse Sum Bui B. Solid-Phase Synthesis of Molecularly Imprinted Nanoparticles for Protein Recognition. Chem. Commun. 2013, 49 (60), 6746–6748. 10.1039/c3cc41701h. 23785709

[ref8] Kunath S.; Panagiotopoulou M.; Maximilien J.; Marchyk N.; Sänger J.; Haupt K. Cell and Tissue Imaging with Molecularly Imprinted Polymers as Plastic Antibody Mimics. Adv. Healthcare Mater. 2015, 4 (9), 1322–1326. 10.1002/adhm.201500145. 25880918

[ref9] Dong Y.; Li W.; Gu Z.; Xing R.; Ma Y.; Zhang Q.; Liu Z. Inhibition of HER2-Positive Breast Cancer Growth by Blocking the HER2 Signaling Pathway with HER2-Glycan-Imprinted Nanoparticles. Angew. Chem., Int. Ed. 2019, 58 (31), 10621–10625. 10.1002/anie.201904860. 31166063

[ref10] Medina Rangel P. X.; Moroni E.; Merlier F.; Gheber L. A.; Vago R.; Tse Sum Bui B.; Haupt K. Chemical Antibody Mimics Inhibit Cadherin-Mediated Cell–Cell Adhesion: A Promising Strategy for Cancer Therapy. Angew. Chem., Int. Ed. 2020, 59 (7), 2816–2822. 10.1002/anie.201910373. 31659849

[ref11] Cenci L.; Guella G.; Andreetto E.; Ambrosi E.; Anesi A.; Bossi A. M. Guided Folding Takes a Start from the Molecular Imprinting of Structured Epitopes. Nanoscale 2016, 8 (34), 15665–15670. 10.1039/C6NR03467E. 27524659

[ref12] Gu Z.; Dong Y.; Xu S.; Wang L.; Liu Z. Molecularly Imprinted Polymer-Based Smart Prodrug Delivery System for Specific Targeting, Prolonged Retention, and Tumor Microenvironment-Triggered Release. Angew. Chem., Int. Ed. 2021, 60, 2663 10.1002/anie.202012956. PMC7898932 33078504

[ref13] Staahl M.; Jeppsson-Wistrand U.; Maansson M. O.; Mosbach K. Induced Stereo- and Substrate Selectivity of Bioimprinted. Alpha.-Chymotrypsin in Anhydrous Organic Media. J. Am. Chem. Soc. 1991, 113 (24), 9366–9368. 10.1021/ja00024a051.

[ref14] Yang S.; Wang Y.; Xu M.; He M.; Zhang M.; Ran D.; Jia X. Synthesis of Modified Chitosan-Based Molecularly Imprinted Polymers for Adsorptive Protein Separation. Anal. Methods 2013, 5 (20), 5471 10.1039/c3ay41000e.

[ref15] Dan R.; Wang Y.; Du L.; Du S.; Huang M.; Yang S.; Zhang M. The Synthesis of Molecular Imprinted Chitosan-Gels Copolymerized with Multiform Functional Monomers at Three Different Temperatures and the Recognition for the Template Ovalbumin. Analyst 2013, 138 (12), 3433 10.1039/c3an36930g. 23640161

[ref16] Herrero E. P.; Martín Del Valle E. M.; Peppas N. A. Protein Imprinting by Means of Alginate-Based Polymer Microcapsules. Ind. Eng. Chem. Res. 2010, 49 (20), 9811–9814. 10.1021/ie101068z.

[ref17] Omenetto F. G.; Kaplan D. L. New Opportunities for an Ancient Material. Science (Washington, DC, U. S.) 2010, 329 (5991), 528–531. 10.1126/science.1188936. PMC3136811 20671180

[ref18] Motta A.; Maniglio D.; Migliaresi C.; Kim H.-J.; Wan X.; Hu X.; Kaplan D. L. Silk Fibroin Processing and Thrombogenic Responses. J. Biomater. Sci., Polym. Ed. 2009, 20 (13), 1875–1897. 10.1163/156856208X399936. 19793445

[ref19] Altman G. H.; Diaz F.; Jakuba C.; Calabro T.; Horan R. L.; Chen J.; Lu H.; Richmond J.; Kaplan D. L. Silk-Based Biomaterials. Biomaterials 2003, 24 (3), 401–416. 10.1016/S0142-9612(02)00353-8. 12423595

[ref20] Nguyen T. P.; Nguyen Q. V.; Nguyen V.-H.; Le T.-H.; Huynh V. Q. N.; Vo D.-V. N.; Trinh Q. T.; Kim S. Y.; Van Le Q. Silk Fibroin-Based Biomaterials for Biomedical Applications: A Review. Polymers (Basel, Switz.) 2019, 11 (12), 1933 10.3390/polym11121933. PMC6960760 31771251

[ref21] Xiao L.; Lu G.; Lu Q.; Kaplan D. L. Direct Formation of Silk Nanoparticles for Drug Delivery. ACS Biomater. Sci. Eng. 2016, 2 (11), 2050–2057. 10.1021/acsbiomaterials.6b00457. 33440541

[ref22] Numata K.; Kaplan D. L. Silk-Based Delivery Systems of Bioactive Molecules. Adv. Drug Delivery Rev. 2010, 62 (15), 1497–1508. 10.1016/j.addr.2010.03.009. PMC2901564 20298729

[ref23] Takeuchi T.; Kitayama Y.; Sasao R.; Yamada T.; Toh K.; Matsumoto Y.; Kataoka K. Molecularly Imprinted Nanogels Acquire Stealth In Situ by Cloaking Themselves with Native Dysopsonic Proteins. Angew. Chem., Int. Ed. 2017, 56 (25), 7088–7092. 10.1002/anie.201700647. 28455941

[ref24] Li G.; Li Y.; Chen G.; He J.; Han Y.; Wang X.; Kaplan D. L. Silk-Based Biomaterials in Biomedical Textiles and Fiber-Based Implants. Adv. Healthcare Mater. 2015, 4 (8), 1134–1151. 10.1002/adhm.201500002. PMC4456268 25772248

[ref25] Min B. M.; Lee G.; Kim S. H.; Nam Y. S.; Lee T. S.; Park W. H. Electrospinning of Silk Fibroin Nanofibers and Its Effect on the Adhesion and Spreading of Normal Human Keratinocytes and Fibroblasts in Vitro. Biomaterials 2004, 25 (7–8), 1289–1297. 10.1016/j.biomaterials.2003.08.045. 14643603

[ref26] Silva S. S.; Maniglio D.; Motta A.; Mano J. F.; Reis R. L.; Migliaresi C. Genipin-Modified Silk-Fibroin Nanometric Nets. Macromol. Biosci. 2008, 8 (8), 766–774. 10.1002/mabi.200700300. 18432596

[ref27] Lammel A. S.; Hu X.; Park S.-H.; Kaplan D. L.; Scheibel T. R. Controlling Silk Fibroin Particle Features for Drug Delivery. Biomaterials 2010, 31 (16), 4583–4591. 10.1016/j.biomaterials.2010.02.024. 20219241 PMC2846964

[ref28] Bucciarelli A.; Muthukumar T.; Kim J. S.; Kim W. K.; Quaranta A.; Maniglio D.; Khang G.; Motta A. Preparation and Statistical Characterization of Tunable Porous Sponge Scaffolds Using UV Cross-Linking of Methacrylate-Modified Silk Fibroin. ACS Biomater. Sci. Eng. 2019, 5 (12), 6374–6388. 10.1021/acsbiomaterials.9b00814. 33417790

[ref29] Qian L.; Hu X.; Guan P.; Wang D.; Li J.; Du C.; Song R. An Effective Way to Imprint Protein with the Preservation of Template Structure by Using a Macromolecule as the Functional Monomer. RSC Adv. 2015, 5 (73), 59062–59069. 10.1039/C5RA08246C.

[ref30] Myers R. H.; Khuri A. I.; Carter W. H. Response Surface Methodology: 1966–L988. Technometrics 1989, 31 (2), 137–157. 10.1080/00401706.1989.10488509.

[ref31] Brown M. P.; Royer C. Fluorescence Spectroscopy as a Tool to Investigate Protein Interactions. Curr. Opin. Biotechnol. 1997, 8 (1), 45–49. 10.1016/S0958-1669(97)80156-5. 9013650

[ref32] Pierce M. M.; Raman C. S.; Nall B. T. Isothermal Titration Calorimetry of Protein-Protein Interactions. Methods 1999, 19 (2), 213–221. 10.1006/meth.1999.0852. 10527727

[ref33] Prozeller D.; Morsbach S.; Landfester K. Isothermal Titration Calorimetry as a Complementary Method for Investigating Nanoparticle–Protein Interactions. Nanoscale 2019, 11 (41), 19265–19273. 10.1039/C9NR05790K. 31549702

[ref34] Huang R.; Lau B. L. T. Biomolecule–nanoparticle interactions: Elucidation of the thermodynamics by isothermal titration calorimetry. Biochim. Biophys. Acta, Gen. Subj. 2016, 1860, 945–956. 10.1016/j.bbagen.2016.01.027. 26851677

[ref35] Guerreiro A. R.; Chianella I.; Piletska E.; Whitcombe M. J.; Piletsky S. A. Selection of imprinted nanoparticles by affinity chromatography. Biosens. Bioelectron. 2009, 24 (8), 2740–2743. 10.1016/j.bios.2009.01.013. 19217769

[ref36] Assfalg M.; Ragona L.; Pagano K.; D’Onofrio M.; Zanzoni S.; Tomaselli S.; Molinari H. The study of transient protein-nanoparticle interactions by solution NMR spectroscopy. Biochim. Biophys. Acta, Proteins Proteomics 2016, 1864 (1), 102–114. 10.1016/j.bbapap.2015.04.024. 25936778

[ref37] Zhang C.; Su Y.; Liang Y.; Lai W. Microfluidic Cloth-Based Analytical Devices: Emerging Technologies and Applications. Biosens. Bioelectron. 2020, 168, 112391 10.1016/j.bios.2020.112391. 32862091

[ref38] Patel K. D.; Kim H.; Knowles J. C.; Poma A. Molecularly Imprinted Polymers and Electrospinning: Manufacturing Convergence for Next-Level Applications. Adv. Funct. Mater. 2020, 30 (32), 2001955 10.1002/adfm.202001955.

[ref39] Boutureira O.; Bernardes G. J. L. Advances in Chemical Protein Modification. Chem. Rev. 2015, 115 (5), 2174–2195. 10.1021/cr500399p. 25700113

[ref40] Rockwood D. N.; Preda R. C.; Yücel T.; Wang X.; Lovett M. L.; Kaplan D. L. Materials Fabrication from Bombyx Mori Silk Fibroin. Nat. Protoc. 2011, 6 (10), 1612–1631. 10.1038/nprot.2011.379. 21959241 PMC3808976

[ref41] Kim S. H.; Yeon Y. K.; Lee J. M.; Chao J. R.; Lee Y. J.; Seo Y. B.; Sultan M. T.; Lee O. J.; Lee J. S.; Yoon S.; Hong I.-S.; Khang G.; Lee S. J.; Yoo J. J.; Park C. H. Precisely Printable and Biocompatible Silk Fibroin Bioink for Digital Light Processing 3D Printing. Nat. Commun. 2018, 9 (1), 1620 10.1038/s41467-018-03759-y. 29693652 PMC5915392

[ref42] R Core Team R: A language and environment for statistical computing. R Foundation for Statistical Computing, Vienna, Austria, 2013. URL http://www.R-project.org.

[ref43] Bucciarelli A.; Chiera S.; Quaranta A.; Yadavalli V. K.; Motta A.; Maniglio D. A Thermal-Reflow-Based Low-Temperature, High-Pressure Sintering of Lyophilized Silk Fibroin for the Fast Fabrication of Biosubstrates. Adv. Funct. Mater. 2019, 29 (42), 1901134 10.1002/adfm.201901134.

[ref44] Bucciarelli A.; Reddy Chandraiahgari C.; Adami A.; Mulloni V.; Lorenzelli L. Precise Dot Inkjet Printing Thought Multifactorial Statistical Optimization of the Piezoelectric Actuator Waveform. Flex. Print. Electron. 2020, 5 (4), 045002 10.1088/2058-8585/abbb7e.

[ref45] Rueden C. T.; Schindelin J.; Hiner M. C.; DeZonia B. E.; Walter A. E.; Arena E. T.; Eliceiri K. W. ImageJ2: ImageJ for the next Generation of Scientific Image Data. BMC Bioinf. 2017, 18 (1), 529 10.1186/s12859-017-1934-z. PMC5708080 29187165

