# Supplementary material for: Molecularly Imprinted Silk Fibroin Nanoparticles
Source: ACS Appl Mater Interfaces. 2021 Jun 30;13(27):31431–9. doi: 10.1021/acsami.1c05405 (PMC8289228; doi:10.1021/acsami.1c05405)
Supplement: Supplementary file 1 — am1c05405_si_001.pdf [file am1c05405_si_001.pdf]

Supporting Information

Molecularly imprinted silk fibroin nanoparticles.

Alessandra Maria Bossi[1], Alessio Bucciarelli[2], Devid Maniglio[3**]*

1. University of Verona, Department of Biotechnology, Strada Le Grazie 15, 37134 Verona, Italy.

2. National Council or Research, CNR-Nanotec, Campus Ecotekne - Università del Salento via Monteroni - 73100 Lecce, Italy

3. University of Trento, Department of Industrial Engineering, BIOtech Research Center, Via delle Regole 101, Mattarello, 38123 Trento, Italy.

Corresponding Authors

* Alessandra Maria Bossi, email: alessandramaria.bossi@univr.it

** Devid Maniglio, email: devid.maniglio@unitn.it

1. **Dynamic light scattering of non-methacrylated silk fibroin nanoparticles. Preliminary experimental evidences over the formation of SF-NPs.**
2. **Surface Response Method**
3. **Scanning electron microscopy images**
4. **Static light scattering for the estimation of molecular weight**
5. **Zeta potential**
6. **Template removal**
7. **Binding of template to MIP SF-NPs**
8. **Isothermal titration nanocalorimetry**
9. **Cell toxicity**
10. **Functional silk fibers decorated with MIP SF-NPs**

1. Dynamic light scattering of non-methacrylated silk fibroin nanoparticles. Preliminary experimental evidences over the formation of SF-NPs.

Figure S1.1 SF-NPs prepared from SF-MA 0.3%. Each measure represents the average of three technical replicas. Red: freshly diluted (Zave=69 nm; PDI 0.329). Green after 4 hours (Zave=65; PDI 0.351)

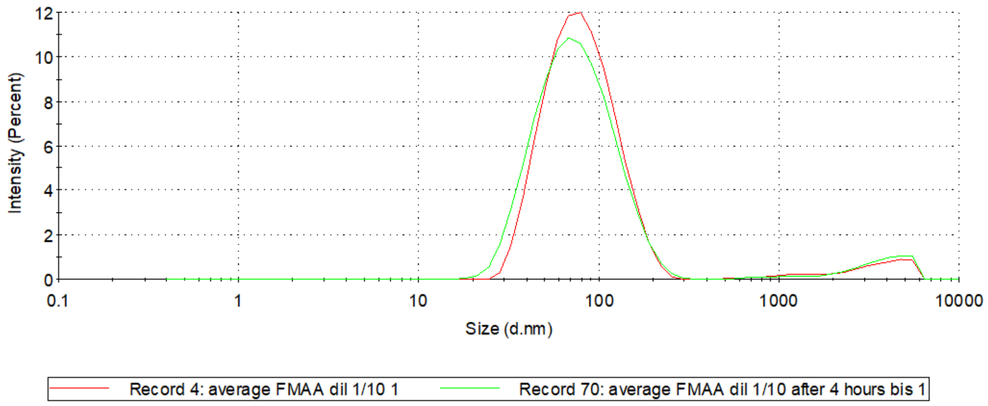

Figure S1.2 SF-NPs prepared from SF-MA 0.03%. Each measure represents the average of three technical replicas. Red: freshly diluted (Zave=63 nm; PDI 0.318). Green after 4 hours (Zave=120; PDI 0.56).

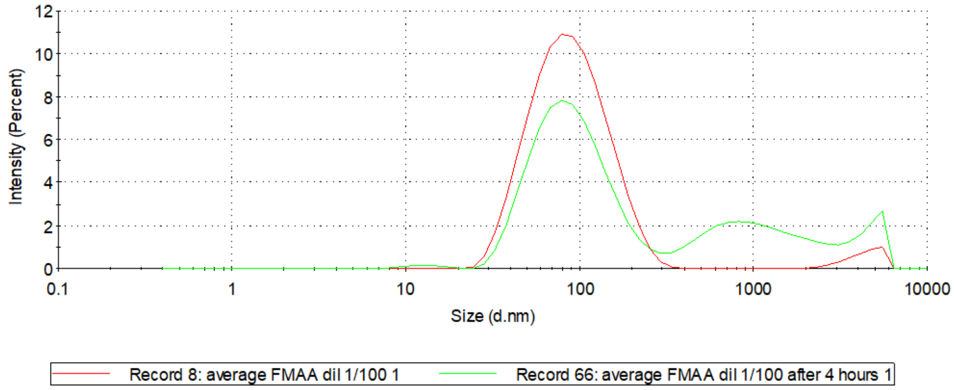

Figure S1.3 SF-NPs prepared from SF-MA 0.3%. Each measure represents the average of three technical replicas. Red: non-polymerized (Z_{ave}=65 nm). Blue: polymerized (Z_{ave} = 79 nm). Green: polymerized; stored overnight (Z_{ave} = 81 nm).

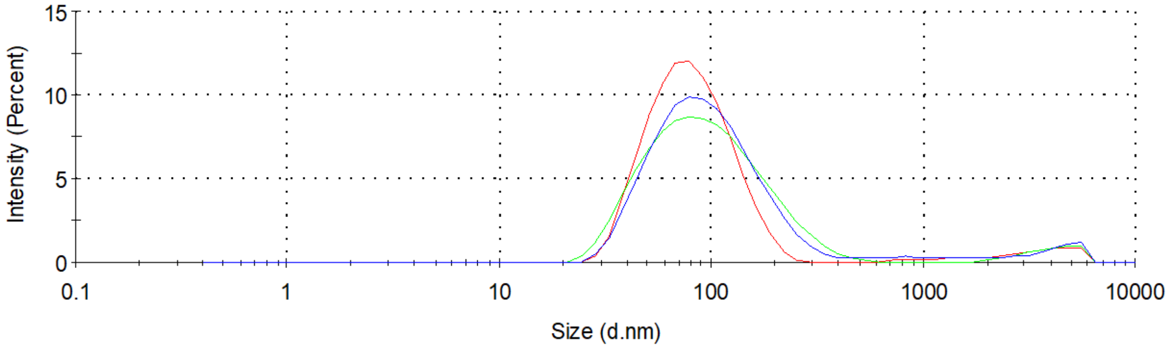

Figure S1.4 SF-NPs prepared from SF-MA 0.03%. Each measure represents the average of three technical replicas. Red: non-polymerized (Z_{ave} = 73 nm). Blue: polymerized (Z_{ave} = 58 nm). Green: polymerized; stored overnight (Z_{ave} = 55 nm).

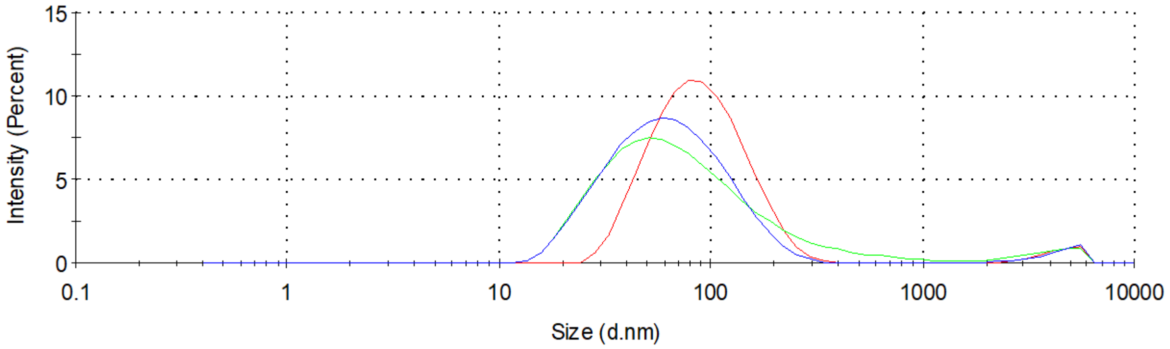

2. Response surface method

Table S2.1: Table reporting the values of the studied parameters and the corresponding mean value of the nanoparticle diameter and its standard deviation.

A<br>pH	B<br>% LAP	C<br>%	diameter<br>nm	PDI
3.5	0.04	0.03	60.6	0.27
3.5	0.04	0.03	60.9	0.27
3.5	0.04	0.03	60.9	0.29
3.5	0.04	0.3	99.3	0.28
3.5	0.04	0.3	99.8	0.26
3.5	0.04	0.3	99.3	0.25
5	0.04	0.03	59.1	0.27
5	0.04	0.03	58.7	0.27
5	0.04	0.03	62.4	0.27
5	0.04	0.3	97.5	0.42
5	0.04	0.3	98.3	0.37
5	0.04	0.3	98.3	0.41
7.4	0.04	0.03	69.6	0.22
7.4	0.04	0.03	68	0.22
7.4	0.04	0.03	70.4	0.22
7.4	0.04	0.3	115	0.87
7.4	0.04	0.3	118.1	0.71
7.4	0.04	0.3	114	0.7
9.8	0.04	0.03	63.5	0.23
9.8	0.04	0.03	62.7	0.24
9.8	0.04	0.03	58.7	0.22
9.8	0.04	0.3	113.3	0.92
9.8	0.04	0.3	96.4	0.86
9.8	0.04	0.3	98.2	0.89
3.5	0.2	0.03	51.3	0.23
3.5	0.2	0.03	51.2	0.23
3.5	0.2	0.03	57.7	0.23
3.5	0.2	0.3	112.2	0.23
3.5	0.2	0.3	90.9	0.22
3.5	0.2	0.3	107	0.23
5	0.2	0.03	58.7	0.18
5	0.2	0.03	58.8	0.19
5	0.2	0.03	57.7	0.18
5	0.2	0.3	100.9	0.24
5	0.2	0.3	103	0.25
5	0.2	0.3	103	0.25
7.4	0.2	0.03	52.3	0.22
7.4	0.2	0.03	52.3	0.13
7.4	0.2	0.03	52	0.13
7.4	0.2	0.3	84	0.28
7.4	0.2	0.3	88	0.25
7.4	0.2	0.3	89.7	0.28
9.8	0.2	0.03	60.9	0.29
9.8	0.2	0.03	60.6	0.16
9.8	0.2	0.03	60.8	0.16
9.8	0.2	0.3	90.9	0.29
9.8	0.2	0.3	91.2	0.29
9.8	0.2	0.3	91.4	0.29

Using the DFBETAS test the highlighted points in **Table S2.1** resulted to be possible outlier. However, as further discussed below, even with the presence of outliers both the regressive models well fit to the data points. To optimize even further the model this 3 point should be repeated.

Table S2.1: Scatter plot matrix with the corresponding Pearson's r indices. More the index is close to 1 more the two considered variables are correlated. An index higher than 0.3 is considered as sign of a significative correlation. The ellipsoid indicates the 95% CI.

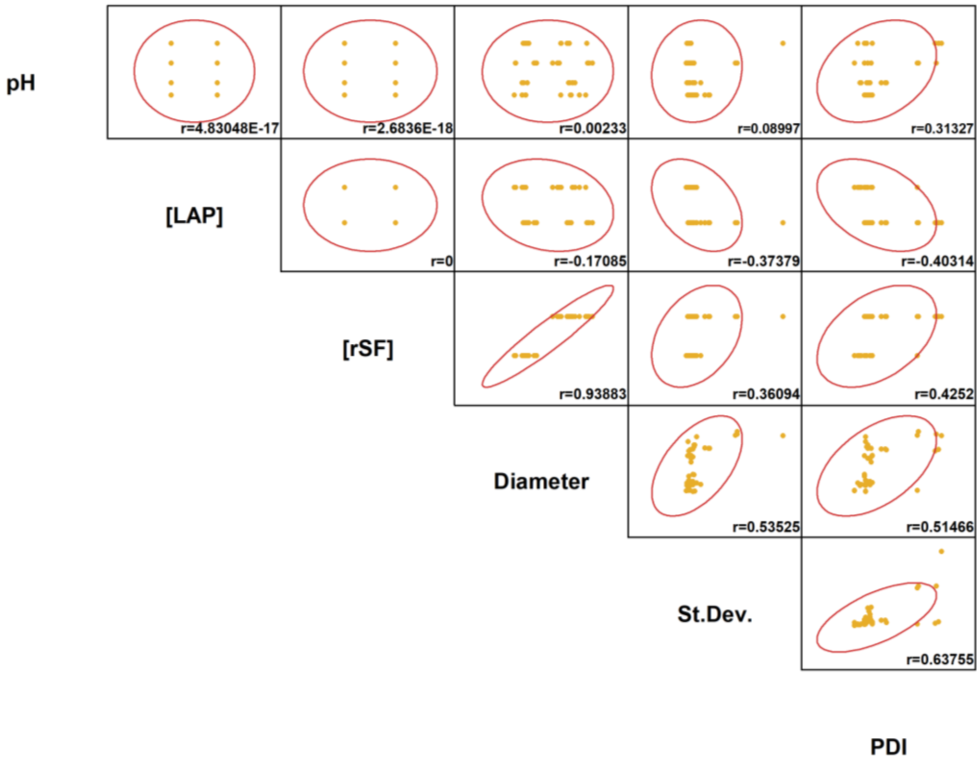

As pre-analysis a scatter plot has been computed, the Pearson's index indicates which factors has the higher impact on the considered property. The diameter results to be strongly dependent on the fibroin concentration. The standard deviation is slightly dependent on both the fibroin and the photoinitiator concentration. The PDI is moderately dependent on the fibroin and photoinitiator concentration and slightly dependent on the pH. It is possible, in addition, to state than a higher diameter implies a higher standard deviation and a higher PDI (in fact both the r indexes are positive and higher than 0.3). Since this method does not provide any information on the dependence of the properties on the mixed factors (like pH*[LAP]), to obtain this kind of information the further data analysis has been performed. The predictive models for the diameter and for the polydispersity index are shown in the **Figure 1A** as contour plots.

Table S2.2: ANOVA table for the predictive model of the nanoparticle diameter. The significance level is assigned as follow: p≤0.1 (.), p≤0.05 (*), p≤0.01 (**), p≤0.001 (***).

**Source**	**Sum of Squares**	**df**	**Mean Square**	**F-value**	**p-value**	
**Model**	0.0116	11	0.0011	194.27	< 0.0001	significant
A-pH	4.737E-09	1	4.737E-09	0.0009	0.9766	***
B-LAP conc.	0.0005	1	0.0005	94.83	< 0.0001	***
C-Fibroin conc.	0.0103	1	0.0103	1895.01	< 0.0001	***
AB	0.0003	1	0.0003	61.45	< 0.0001	***
AC	0.0001	1	0.0001	13.24	0.0009	***
BC	0.0000	1	0.0000	4.60	0.0388	*
A^2	5.835E-06	1	5.835E-06	1.08	0.3064	
ABC	0.0001	1	0.0001	14.29	0.0006	***
A^2B	0.0001	1	0.0001	23.80	< 0.0001	***
A^3	6.096E-07	1	6.096E-07	0.1125	0.7393	
A^3B	0.0003	1	0.0003	54.14	< 0.0001	***
**Residual**	0.0002	36	5.419E-06			
Lack of Fit	0.0000	4	4.425E-06	0.7982	0.5353	not significant
Pure Error	0.0002	32	5.544E-06			
**Cor Total**	0.0118	47				

The ANOVA test of the diameter predictive model is shown in **Table S2.2**. The **Model F-value** of 194.27 implies that it is significant (there is only a 0.01% chance that an F-value this large could occur due to noise), **P-values** less than 0.0500 indicate model terms are significant. In this case A, C, AB, BC, A^2, A^2B, A^2C, A^3, A^3B are significant model terms, the other terms were added to maintain the model hierarchy. The **Lack of Fit F-value** of 0.80 implies the Lack of Fit is not significant relative to the pure error (there is a 53% chance that a Lack of Fit F-value this large could occur due to noise). The model results to well fit the data in fact, as can be clearly seen from the predicted versus actual graph in **Figure S2.1**, the points are close to the line. All the coefficients of the predictive equations including the 95% CI are reported in **Table S2.3**.

Figure S2.1: Predicted versus Actual graph for the nanoparticle diameter model. The model well fits the collected data, in fact, the points relative to the data are close to the line, that represent a perfect fitting.

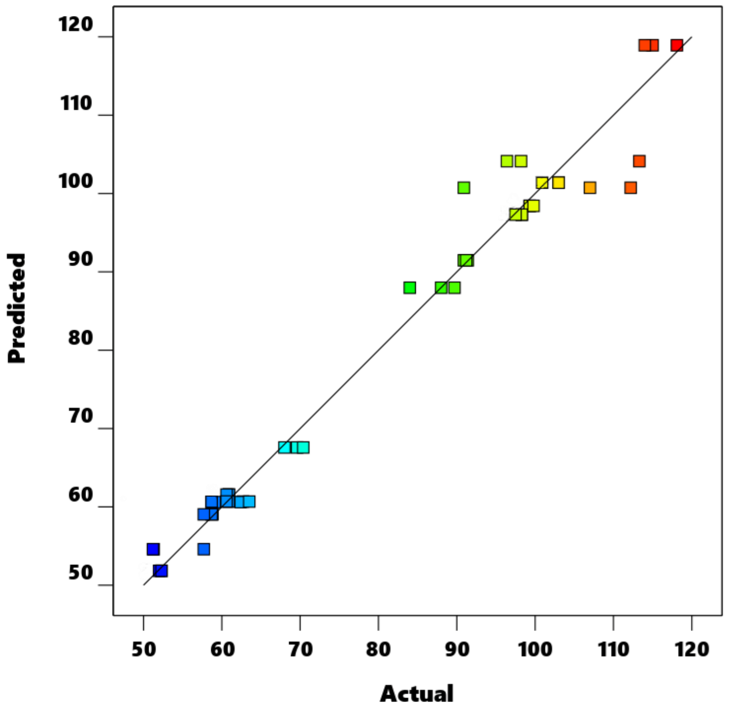

Table S.2.3: Estimation of the model and the 95% CI coefficients of the nanoparticle diameter. The equations are in the coded form (with all the factors varying between -1 and 1).

Factor	Coefficient Estimate	df	Standard Error	95% CI Low	95% CI High
Intercept	0.1146	1	0.0006	0.1135	0.1158
A-pH	-0.0000	1	0.0016	-0.0032	0.0031
B-LAP conc.	0.0056	1	0.0006	0.0044	0.0068
C-Fibroin conc.	-0.0147	1	0.0003	-0.0154	-0.0140
AB	0.0122	1	0.0016	0.0090	0.0153
AC	0.0016	1	0.0004	0.0007	0.0025
BC	-0.0007	1	0.0003	-0.0014	-0.0000
A^2	0.0008	1	0.0008	-0.0008	0.0024
ABC	0.0017	1	0.0004	0.0008	0.0026
A^2B	-0.0039	1	0.0008	-0.0055	-0.0023
A^3	-0.0006	1	0.0017	-0.0039	0.0028
A^3B	-0.0122	1	0.0017	-0.0156	-0.0089

Table S2.4: ANOVA table for the predictive model of the nanoparticle polydispersity index. The significance level is assigned as follow: p≤0.1 (.), p≤0.05 (*), p≤0.01 (**), p≤0.001 (***).

Source	Sum of Squares	df	Mean Square	F-value	p-value	
**Model**	1.02	10	0.1020	106.09	< 0.0001	significant
A-pH	0.0766	1	0.0766	79.73	< 0.0001	***
B-LAP conc.	0.1433	1	0.1433	149.11	< 0.0001	***
C-Fibroin conc.	0.1891	1	0.1891	196.80	< 0.0001	***
AB	0.0585	1	0.0585	60.84	< 0.0001	***
AC	0.1577	1	0.1577	164.13	< 0.0001	***
BC	0.1028	1	0.1028	106.93	< 0.0001	***
A^2	3.774E-06	1	3.774E-06	0.0039	0.9504	
ABC	0.0726	1	0.0726	75.58	< 0.0001	***
A^2B	0.0070	1	0.0070	7.28	0.0105	*
A^2C	0.0144	1	0.0144	14.94	0.0004	***
**Residual**	0.0356	37	0.0010			
Lack of Fit	0.0060	5	0.0012	1.31	0.2851	not significant
Pure Error	0.0295	32	0.0009			
**Cor Total**	1.06	47				

The ANOVA test of the PDI model is shown in **Table S2.5**. The **Model F-value** of 106.09 implies the model is significant (there is only a 0.01% chance that an F-value this large could occur due to noise). **P-values** less than 0.0500 indicate model terms are significant. In this case A, B, C, AB, AC, BC, ABC, A^2B, A^2C are significant model terms, the other terms were added to maintain the model hierarchy Values greater than 0.1000 indicate the model terms are not significant. The **Lack of Fit F-value** of 0.28 implies the Lack of Fit is not significant relative to the pure error (there is a 28.29% chance that a Lack of Fit F-value this large could occur due to noise). The model results to well fit the data in fact, as can be clearly seen from the predicted versus actual graph in **Figure S2.2**, the points are close to the line. All the coefficients of the predictive equations including the 95% CI are reported in **Table S2.6**.

Figure S2.2: Predicted versus Actual graph for the standard deviation of the nanoparticle polydispersity index. The model well fits the collected data, in fact, the points relative to the data are close to the line, that represent a perfect fitting.

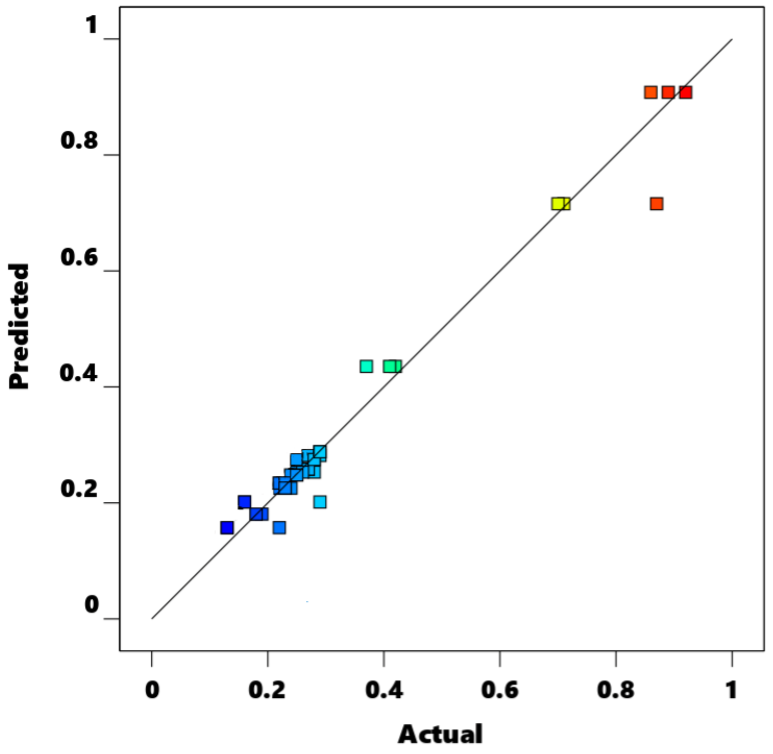

Table S2.5: Estimation of the model and the 95% CI coefficients of the nanoparticle polydispersity index. The equations are in the coded form (with all the factors varying between -1 and 1).

Factor	Coefficient Estimate	df	Standard Error	95% CI Low	95% CI High
Intercept	0.5493	1	0.0076	0.5338	0.5647
A-pH	0.0526	1	0.0059	0.0407	0.0645
B-LAP conc.	-0.0931	1	0.0076	-0.1086	-0.0777
C-Fibroin conc.	0.1070	1	0.0076	0.0915	0.1224
AB	-0.0459	1	0.0059	-0.0579	-0.0340
AC	0.0755	1	0.0059	0.0635	0.0874
BC	-0.0465	1	0.0045	-0.0556	-0.0374
A^2	0.0007	1	0.0106	-0.0207	0.0220
ABC	-0.0512	1	0.0059	-0.0631	-0.0393
A^2B	0.0285	1	0.0106	0.0071	0.0498
A^2C	-0.0408	1	0.0106	-0.0622	-0.0194

3. **Scanning electron microscopy images**

Selected images of the SF-NPs.

Figure S3.1. Upper row: SF-NPs prepared from 0.3% w/w SF starting material prepared at pH 7.0 with LAP 0.04% w/v (left) and LPA 0.2% (right).
Lower row: SF-NPs from, prepared from 0.03% w/w SF starting material prepared at pH 7.0 with LAP 0.04% (left) and LAP 0.2% w/v (right).

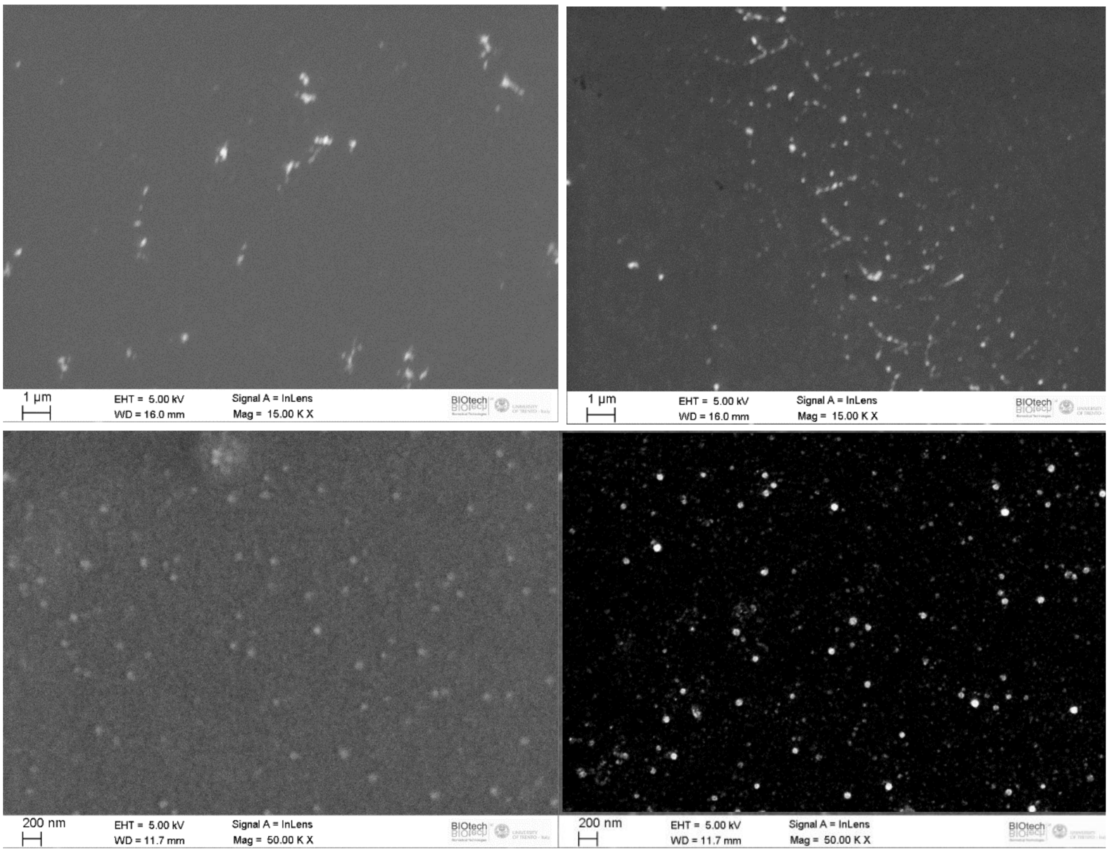

Figure S3.2. SEM image of MIP SF-NPs prepared from 0.3% w/w SF starting material.

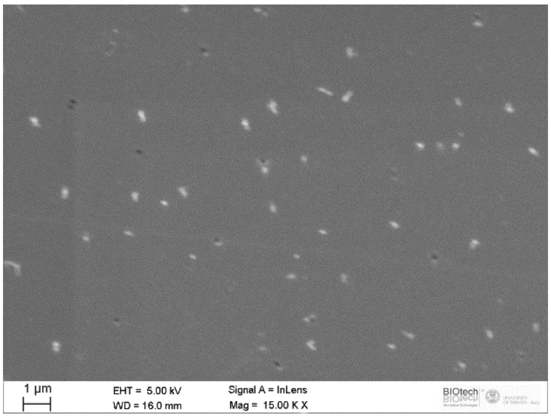

4. Static light scattering for the estimation of molecular weight

Figure S4.1 SF NPs from 0.3% v/w SF-MA had an estimated M_n of 21 ± 3 MDa.

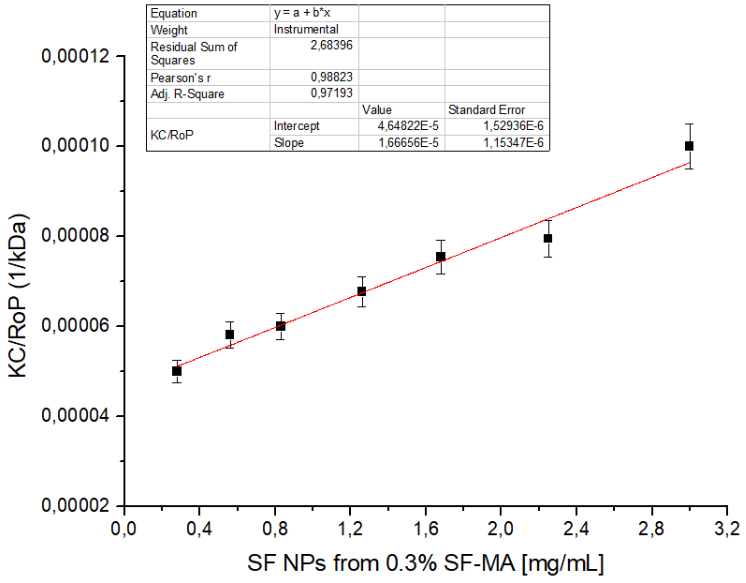

Figure S4.2 SF NPs from 0.03% v/w SF-MA had an estimated M_n of 7.2 ± 1.3 MDa.

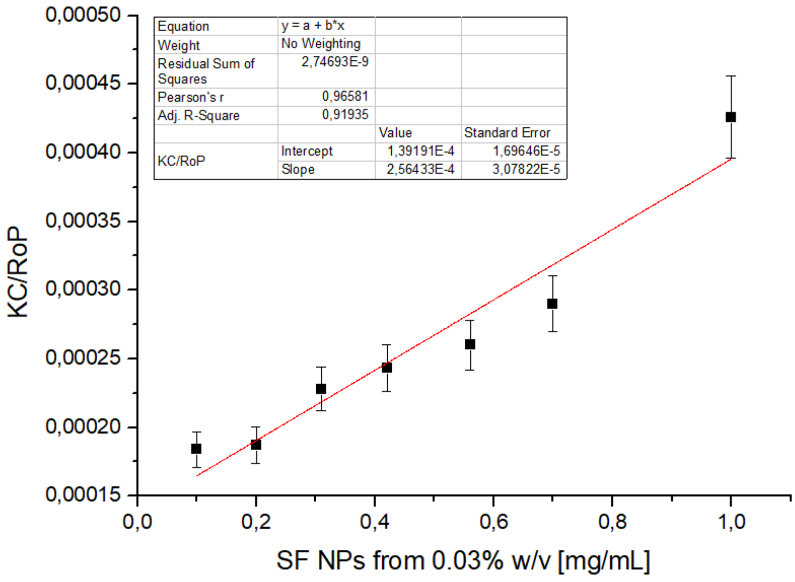

5. Zeta potential

Figure S5.1. Zeta potential of SF-NPs.

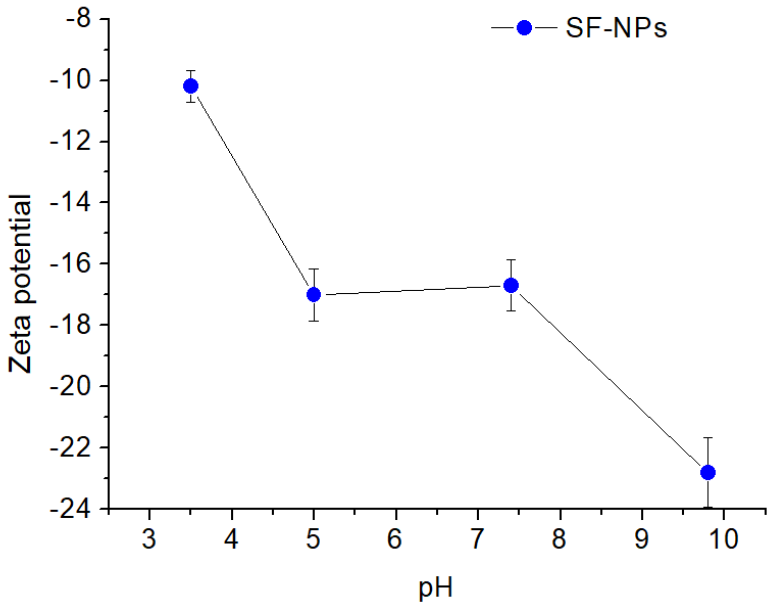

6. Template removal

The MIP SF-NPs were synthesized from 0.3% w/v SF-MA in PB pH 7.4 5 mM and using as a template HSA (200 µg, V= 4 mL final) LAP 0.2%. Photopolymerized for 10 min.

A1 mL of MIP SF-NPs was treated with Triza base 50 mM (100 uL for 1mL, pH 10), let stand 1 hour, then divided in 2 x 500 µL and microfuged on 100 KDa MWCO (MerckMillipore, Darmstadt, Germany) for 4 times at 10.000 x g (addition of 200 µL of PB pH 7.4 Tween 0.05% each time).

Another 1 mL was treated with trypsin (80 µg), let stand 2 hours at room temperature, then divided in 2 x 500 µL and microfuged on 100 KDa MWCO for 4 times at 10.000 x g (addition of 200 µL of PB pH 7.4 Tween 0.05% each time).

Each sample (20 uL) was admixed with loading buffer 4X (10 µL) and loaded onto precast 4-20% gradient SDS gel (BioRad, Hercules, CA, US). The running conditions were 12 mA/gel 15 min; 24 mA/gel (max 7W) 45 min. Staining: Coomassie G250 (Sigma Aldrich), ethanol, acetic acid. Destaining: ethanol, acetic acid, water.

Figure S6.1 SDS PAGE of MIP SF-NP after template removal.

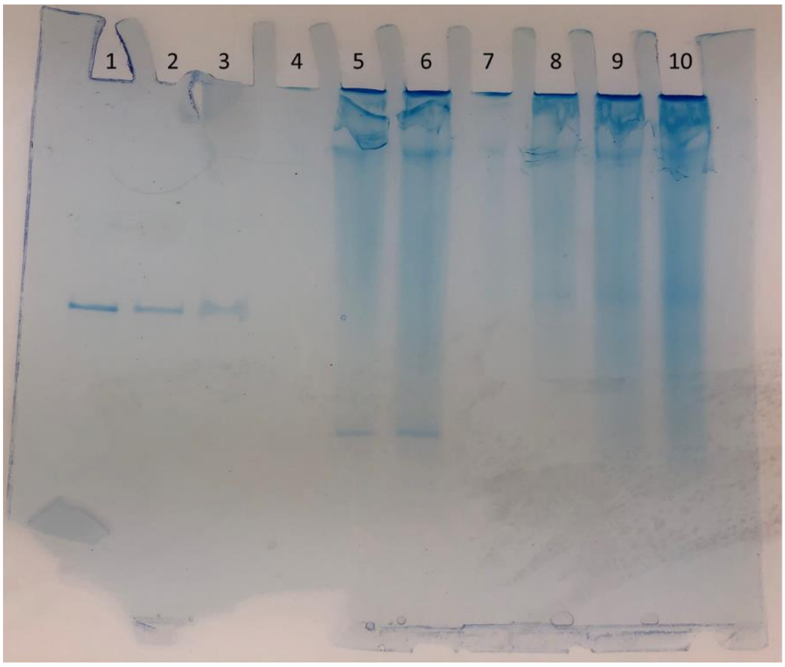

1: HSA 0.5µg
2: HSA 0.25µg
3: HSA 0.1µg
4: -
5: MIP SF-NPs trypsin treated
6: MIP SF-NPs trypsin treated
7: -
8: MIP SF-NPs Tris treated
9: MIP SF-NPs Tris treated
10: MIP SF-NPs Tris treated

The lack of albumin in the SDS PAGE gel indicated the effectiveness of the washings and the absence of loose template protein in the MIPs. Trypsin was chosen as protocol for albumin removal, because it is demonstrated not to degrade SF[1]. To complete the trypsin removal, treated MIP SF-NPs were dialyzed in 5 mM PB pH 7.4 for 4 hours on 50 kDa m.w.c.o. membranes.

7. Binding of template to MIP SF-NPs

Calibration curve for albumin-FITC

For the calibration curve, fitc-alb was diluted in the range of concentrations from 1 nM to 2 µM. Measurements were performed on a Tecan Infinite PRO 200 spectrofluorimeter for microplates (Tecan Lifesciences, Switzerland) in triplicate, using 96 Flat Bottom Black Polystyrene microtiter plates (Nunc™ MicroWell™ 96-Well, Nunclon Delta-Treated, Flat-Bottom Microplate, Thermo Scientific, Germany). The excitation was at the λ_{exc}=488 nm and emission was recorded in the range 514-540 nm. Maximum λ_{em} was at 524 nm. Linear regression of datapoints: y (RFU) = 501537 x (nmol) + 155.32 with R^2 = 0.9987.

Increment in fluorescence for the binding of template; reproducibility of batch to batch binding; controls of no binding

Fitc-alb was used to test the binding of MIP-alb SF-NPs. Measurements were performed in triplicate on 96 Flat Bottom Black Polystyrene microtiter plates (Thermo Scientific, Germany). Wells were loaded with 200 µg of MIP-alb SF-NPs and incubated with 30 pmol of fitc-alb in PB 50 mM pH 7.4 supplemented with 0.05% Tween-20 (V_{tot} = 250 µL; V in each well 60 µL). The excitation was at the λ_{exc} = 488 nm and emission was recorded in the range 514-540 nm. Maximum λ_{em} was at 524 nm.

Two batches of independently synthesized MIP-alb NPs were compared. As a control the fluorescence of MIP-alb was tested. As another control, the binding was performed in Trizma base at pH=10 so to unfavor the interaction.

Figure S7.1. MIP-alb (yellow) did confirm very limited fluorescence emission over the recorded spectrum. Binding of fitc-alb to MIP-alb SF-NPs showed a fairly reproducible fluorescent signal (blue and black). The fluorescence was limited in the case of using an alkaline pH to unfavor the interaction (red).

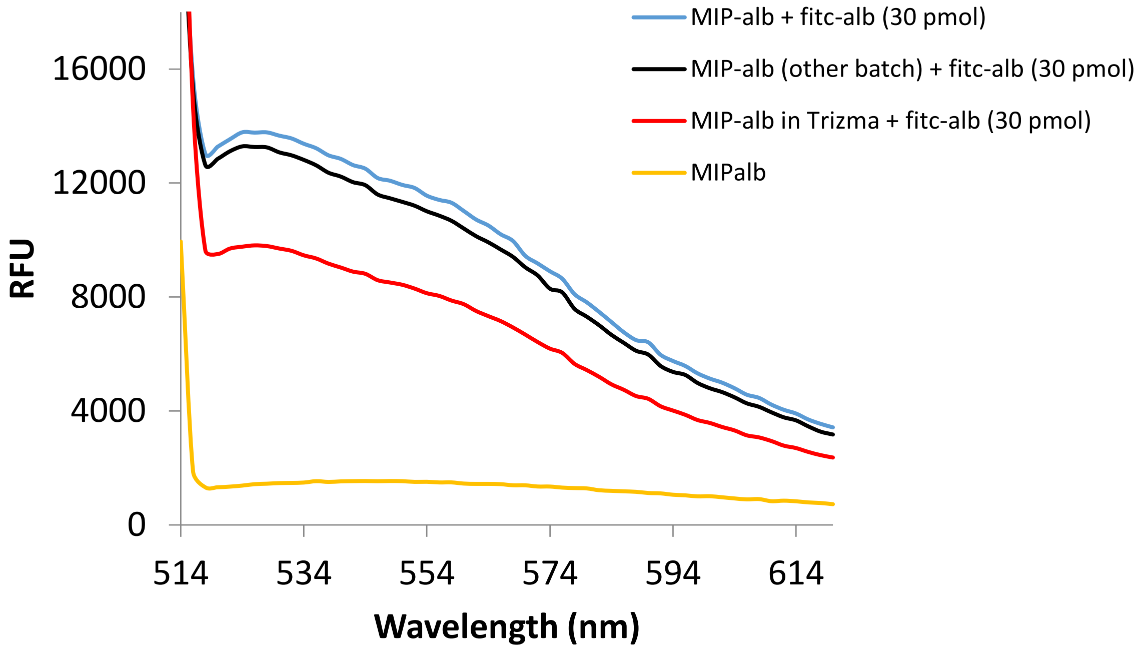

Binding to MIP or to NIP SF-NPs

Figure S7.2. In red: MIP-alb SF-NPs bound to fitc-alb (30 pmol), that results in an enhancement in fluorescence, respect to the fitc-alb (dotted black line). When the digesting enzyme trypsin was added to the fitc-alb bound to MIP-alb, fitc-alb was presumably digested and the emitted fluorescence dropped (light blue). The enhancement of the fluorescence signal was therefore correlated to the binding event. Moreover, the specificity of the binding was supported by the equally low fluorescent signal observed for non imprinted SF-NPs (NIP, purple) in the presence of fitc-alb (30 pmol). As a further control, a MIP SF-NPs prepared using as a template a peptide non-albumin related (green) in the presence of fitc-alb (30 pmol) did show comparable low fluorescence emission to NIP. As control: fluorescence spectra of MIP-alb (yellow).

These results demonstrate the binding of fitc-alb occurred specifically on the MIP-alb and not on the non-imprinted SF-NPs, nor on MIP SF-NPs with non-albumin specific binding sites.

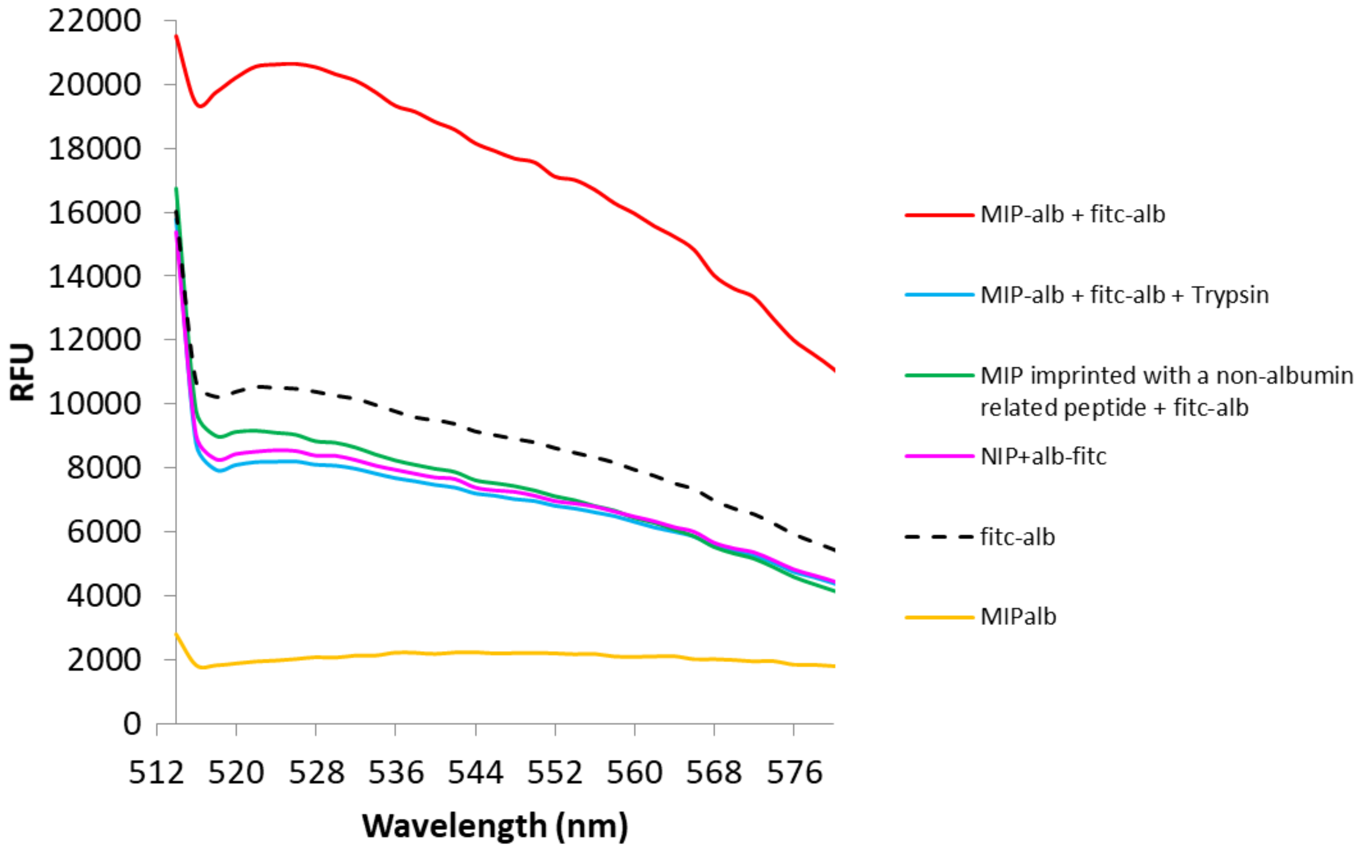

Competitive binding

Figure S7.3. Competitive assay. In red: MIP-alb SF-NPs bound to fitc-alb (30 pmol). When the non fluorescent albumin was added, this resulted in a progressive decrease of the emitted fluorescence (light blue addition of alb 30 pmol; dark blue addition of alb 300 pmol). The decrease resulted more marked for greater quantity of alb competitor, suggesting a displacement of the fitc-alb by alb. As controls fluorescence spectra of MIP-alb (yellow) and fitc-alb 30 pmol (dotted black line).

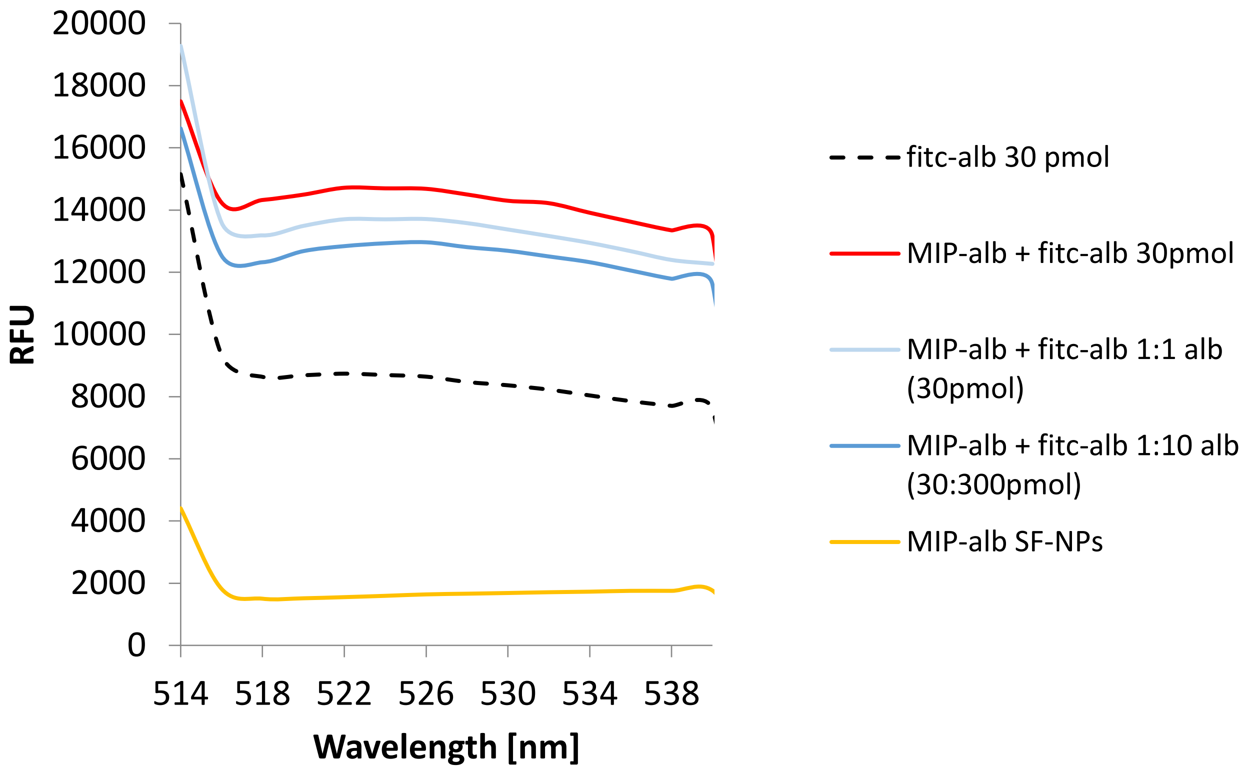

8. Isothermal titration nanocalorimetry

Figure S8.1 Exemplificative raw ITC profiles of MIP-alb SF NPs titrated with the template (HSA, black line) or with non template proteins (cytochrome c, blue line; human transferrin, purple line).

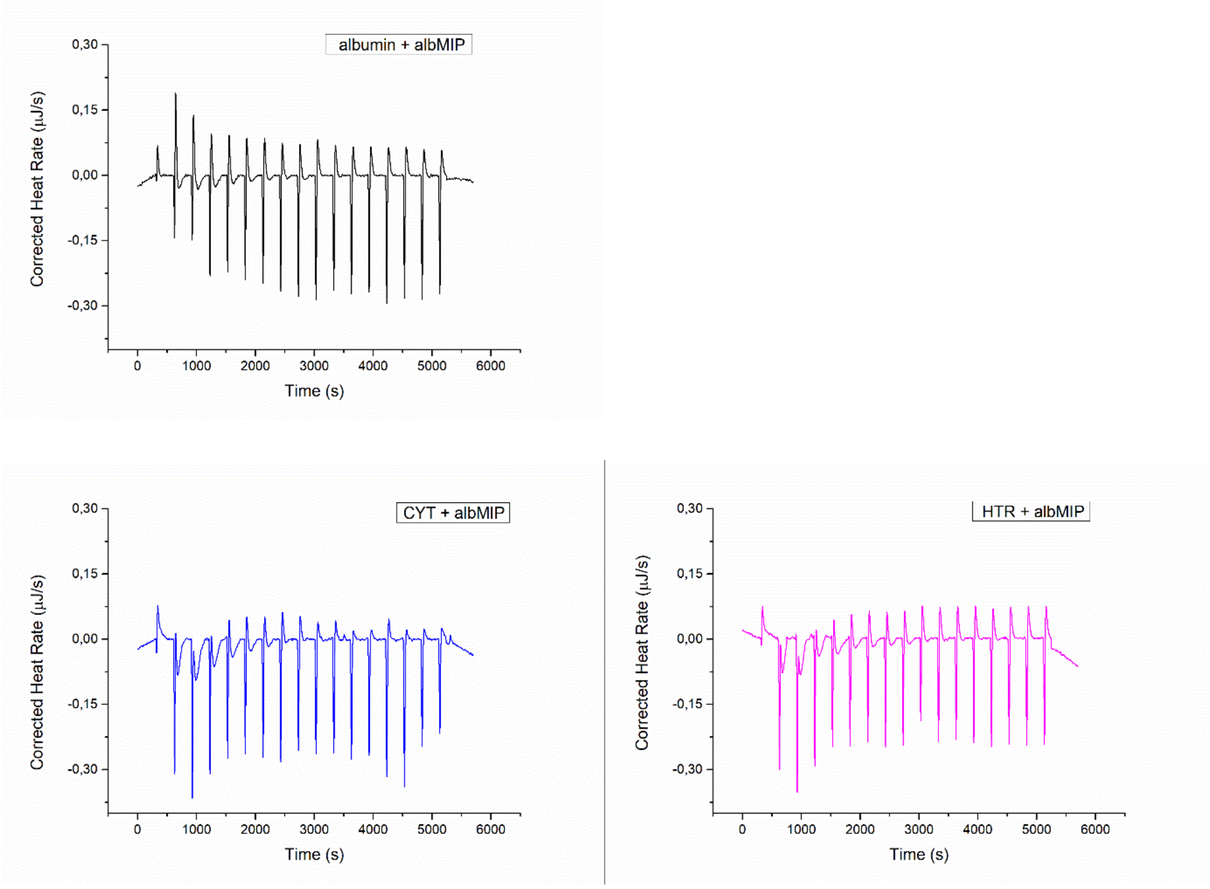

9. Cell toxicity

Figure S9.1. Cell toxicity assay. Cell death percentage of untreated cells (negative control, best viability condition) compared with percentages of cells exposed to 0.25 and 1.5 mg/mL nanoparticle suspension are statistically non-significant ($p > 0.05$, in the specific $p = 0.24$ and $p = 0.137$ respectively). Positive control represents the condition of total cell death.

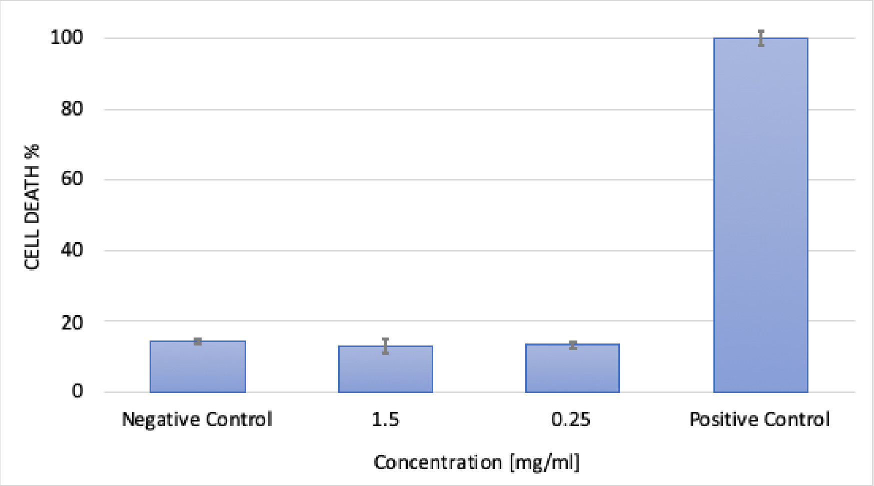

10. Functional silk fibers decorated with MIP SF-NPs

Figure S10.1. Upper panels (Rhod) report the emissions at λ_{em} = 525/50 nm; the lower panels (fitc) report the emissions at λ_{em} = 595/50 nm. Silk fibers (right) and electrospun silk nanofibers (left) coupled to Rhodamine non imprinted SF-NPs (Rhod NIP SF-NPs) showed red emission, confirming a uniform decoration (a-c). Rhod NIP SF-NPs challenged with fitc-alb showed weak fitc signal (b-d) addressable to non-specific albumin adsorption to fibers.

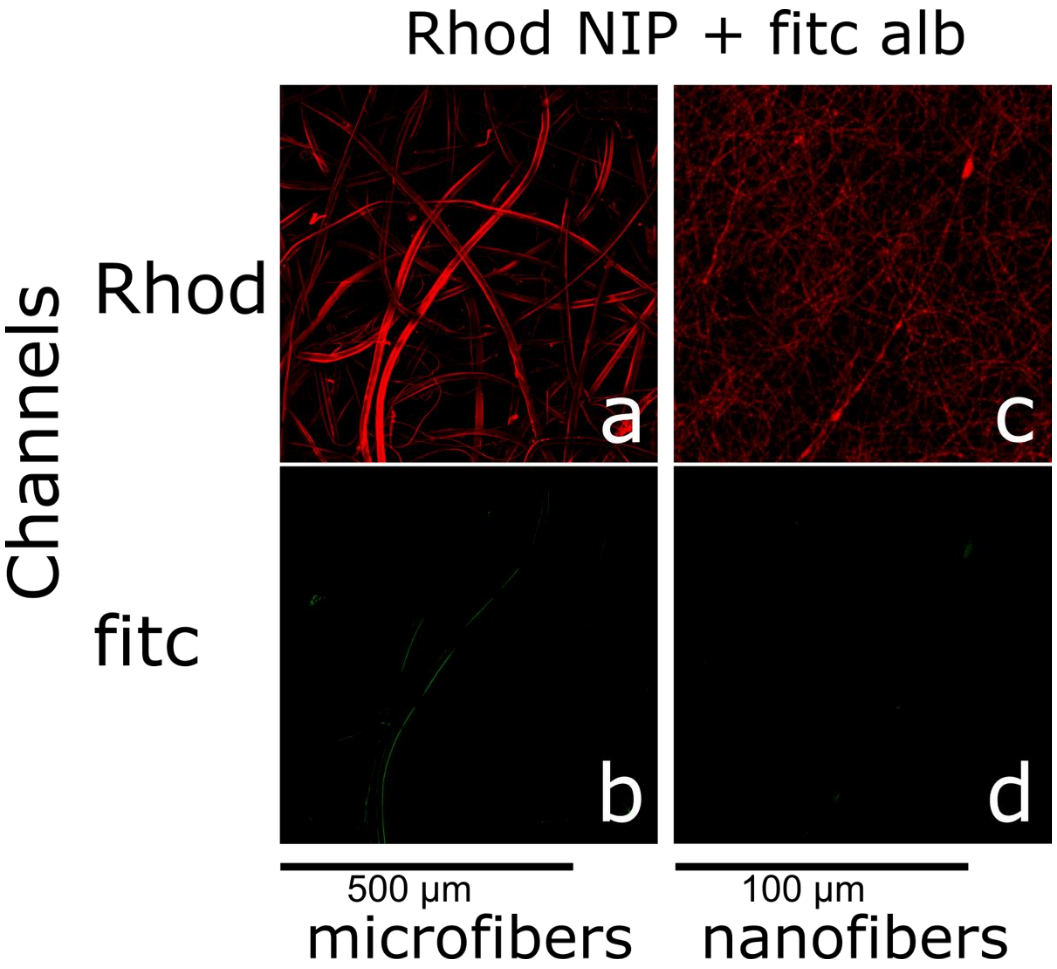

References

1. Horan, R. L.; Antle, K.; Collette, A. L.; Wang, Y.; Huang, J.; Moreau, J. E.; Volloch, V.; Kaplan, D. L.; Altman, G. H. In Vitro Degradation of Silk Fibroin. *Biomaterials* **2005**, *26* (17), 3385–3393. https://doi.org/10.1016/j.biomaterials.2004.09.020.
